# An Update on Development of Small-Molecule Plasmodial Kinase Inhibitors

**DOI:** 10.3390/molecules25215182

**Published:** 2020-11-07

**Authors:** Chantalle Moolman, Rencia van der Sluis, Richard M. Beteck, Lesetja J. Legoabe

**Affiliations:** 1 Centre of Excellence for Pharmaceutical Sciences, North-West University, Private Bag X6001, Potchefstroom 2520, South Africa; 21204756@nwu.ac.za (C.M.); 25159194@nwu.ac.za (R.M.B.); 2 Focus Area for Human Metabolomics, Biochemistry, North-West University, Private Bag X6001, Potchefstroom 2520, South Africa; 21224919@nwu.ac.za

**Keywords:** malaria, *Plasmodium*, kinases, kinase inhibitors, antimalarial agents

## Abstract

Malaria control relies heavily on the small number of existing antimalarial drugs. However, recurring antimalarial drug resistance necessitates the continual generation of new antimalarial drugs with novel modes of action. In order to shift the focus from only controlling this disease towards elimination and eradication, next-generation antimalarial agents need to address the gaps in the malaria drug arsenal. This includes developing drugs for chemoprotection, treating severe malaria and blocking transmission. Plasmodial kinases are promising targets for next-generation antimalarial drug development as they mediate critical cellular processes and some are active across multiple stages of the parasite’s life cycle. This review gives an update on the progress made thus far with regards to plasmodial kinase small-molecule inhibitor development.

## 1. Introduction

Although significant progress has been made with regards to worldwide malaria control and eradication, this infectious disease continues to have devastating effects, especially in developing countries. An estimated 228 million malaria cases and 405,000 malaria-related deaths were reported globally in 2018 [1]. The ongoing coronavirus (COVID-19) pandemic could also have a profound negative impact on the progress made thus far in the fight against malaria [2]. Patients presenting with fever or requiring malaria treatment are now less likely to visit health care facilities out of fear of contracting COVID-19 [3]. Lockdown periods have disrupted the supply of malaria rapid diagnostic tests, antimalarial drugs and other interventions [3]. A number of antimalarial drugs (e.g., artemisinin, chloroquine and hydroxychloroquine) have also been repurposed for COVID-19 treatment, despite a lack of scientific evidence and approval [3]. This has resulted in a shortage of these drugs and, in the long-run, an increased risk of drug resistance due to improper use of antimalarial monotherapies [3].

Apart from the potential impact of the pandemic, recurring antimalarial drug resistance poses a major threat to malaria control and elimination. *P. falciparum*, the *Plasmodium* species responsible for the majority of malaria-related deaths, is resistant to most antimalarial drugs, including the current first-line artemisinin-based combination therapies [1,4]. The second most common human malaria species, *P. vivax*, has developed widespread resistance to chloroquine [5]. Malaria parasites can also develop cross-resistance to antimalarial drugs from the same chemical class or with the same mode of action, which further exacerbates the problem [6].

The life cycle of the malaria parasite is complex; it consists of an asexual stage which occurs in the human host and a sexual stage which occurs in the mosquito vector [7]. Existing antimalarial drugs are highly stage-specific, with the majority targeting blood-stage parasites for treatment of symptomatic malaria [7]. In order to eliminate and eventually eradicate malaria, the focus needs to shift from mainly providing curative treatment to blocking disease transmission [8]. Achieving this goal requires a new generation of cost-effective antimalarial agents that are safe and well-tolerated in a wide range of recipients, including vulnerable populations such as pregnant women and infants [8]. Since drug development is expensive and can take up to 15 years before reaching the market, it is important to have clear guidelines [8]. The Medicines for Malaria Venture (MMV) published defined criteria for the types of individual molecules (target candidate profiles (TCPs)) and drug formulations (target product profiles (TPP)) that would be ideal for new malaria therapy [8]. TPP-1 focusses on treating malaria infections and includes a combination of molecules with blood-stage activity (TCP-1), transmission-blocking activity (TCP-5) and activity against relapse causing liver stages (TCP-3) [8]. Alternatively, TPP-1 could also consist of rapid-acting TCP-1 molecules when treating severe/complicated malaria [8]. TPP-2 focusses on chemoprevention of travelers to endemic regions or during epidemics and includes molecules with TCP-1 and hepatic schizont activity (TCP-4) [8].

Plasmodial kinases have been explored as targets for next-generation antimalarial agents due to their involvement in various critical cellular processes throughout the life cycle of the parasite [9,10,11]. The *P. falciparum* kinome is predicted to encode 85 to 99 protein kinase genes [12,13] as well as a small number of lipid kinase genes. Overall, the *P. falciparum* kinome displays significant divergence from the eukaryotic kinome. The 65 plasmodial kinases that cluster within established eukaryotic protein kinase (ePK) groups (CAMK, AGC, CMGC, CK1 and TKL groups) often display structural and functional characteristics that are not seen in their mammalian counterparts [9,13]. The plasmodial kinome also contains protein kinases that have no mammalian orthologues (orphan kinases) or display homology with more than one of the established ePK groups (composite or hybrid kinases) [9]. These differences can be exploited for selective antimalarial drug development.

Progress on plasmodial kinase inhibitor development up until the beginning of 2018 was discussed in a detailed review by Cabrera and co-workers [14]. Herein, we give an overview thereof, as well as discussing additional kinases and new research related to plasmodial kinase inhibitor development.

## 2. Calcium-Dependent Protein Kinases (CDPKs)

Enzymes from the classical Ca^2+^/calmodulin-dependent protein kinase (CaMK) group seem to be rare in the *P. falciparum* kinome [15]. However, the kinome contains calcium-dependent protein kinases (CDPKs) which have a C-terminal calmodulin-like domain that is highly homologous to the CaMK group [16]. CDPKs comprise a unique family of serine/threonine kinases only found in plants, protozoans (including apicomplexan parasites) and some algae [17]. These enzymes play an important role in calcium signalling during the various life stages of the *Plasmodium* parasite [15]. Seven members of the CDPK family (*Pf*CDPK1 to *Pf*CDPK7) have been identified in *P. falciparum* [16]. *Pf*CDPK1 is expressed at all stages of the *Plasmodium* parasite life cycle. During asexual parasite development, *Pf*CDPK1 plays a role in parasite motility [18,19], microneme secretion and subsequent erythrocyte invasion [20], as well as merozoite egress from mature schizonts [19]. Previous studies have shown that *Pf*CDPK1 is likely to be essential for asexual development [19,21,22]; however, the parasite might have other mechanisms in place to compensate for loss of *Pf*CDPK1 activity [21,23,24]. During the sexual development of the parasite, *Pf*CDPK1 activity is indispensable for gametogenesis and subsequent infection of the mosquito vector [21]. In addition, the *P. berghei* homologue (*Pb*CDPK1) is also involved in ookinete development [25].

*Pf*CDPK2, *Pf*CDPK3 and *Pf*CDPK4 are all predominantly expressed during the sexual stage of the parasite development [26,27,28]. *Pf*CDPK2 and *Pf*CDPK4 are essential for male gametocyte exflagellation and transmission to the mosquito vector [26,27]. In addition, *Pf*CDPK4 is also required for sporozoite invasion of hepatocytes [29]. Although the exact function of *Pf*CDPK3 is not yet known, its *P. berghei* orthologue (*Pb*CDPK3) is expressed in ookinetes where it regulates motility required for invading the midgut of the mosquito [30,31].

*Pf*CDPK5 and *Pf*CDPK7 are expressed during the asexual erythrocytic stage. *Pf*CDPK5 acts synergistically with *P. falciparum* protein kinase G (*Pf*PKG) to regulate microneme secretion which is required for merozoites to egress mature schizonts [32,33]. Although *Pf*CDPK5 is essential, the parasite is able to compensate for loss of *Pf*CDPK5 activity through hyperactivation of *Pf*PKG [32]. While CDPK7 is not essential to parasite viability, it still plays an important role in the development of the erythrocytic parasite. The growth rate of CDPK7 knockout parasites is significantly reduced due to a delay in maturation of ring-stage parasites to trophozoites and the release of fewer merozoites from each schizont [34]. Little is currently known about CDPK6 of *P. falciparum*. The *P. berghei* orthologue (*Pb*CDPK6) signals to sporozoites when to stop migration and initiate invasion of hepatocytes [35].

### 2.1. Inhibitor Development for the CDPK Group

The CDPKs are promising drug targets for the development of new antiplasmodial agents as there are no CDPK orthologues in the human host [16]. A unique structural feature of many parasitic CDPKs is the small gatekeeper residue at the hinge region [36]. A small gatekeeper residue results in enlargement of the hydrophobic pocket that accommodates the ATP purine group in the ATP-binding site [36]. Various medicinal chemistry campaigns have developed small-molecule inhibitors termed bumped kinase inhibitors (BKIs) that contain a bulky C3-aryl substituent that can occupy this enlarged hydrophobic pocket [36]. Most mammalian kinases have larger gatekeeper residues that block access to the bulky substituent of BKIs, therefore allowing better selectivity towards the parasitic kinases [36]. Amongst *P. falciparum* CDPKs, *Pf*CDPK4 has the smallest gatekeeper, which is a serine residue, followed by *Pf*CDPK1 with a medium threonine gatekeeper residue [37]. *Pf*CDPK2 (methionine), *Pf*CDPK3 (methionine) and *Pf*CDPK5 (leucine) all have bulky gatekeeper residues [37]. *P. falciparum* CDPK inhibitor development has mainly focussed on *Pf*CDPK1 and *Pf*CDPK4, and most of these inhibitors are BKIs [14].

#### 2.1.1. *Pf*CDPK1

**Figure 1:**
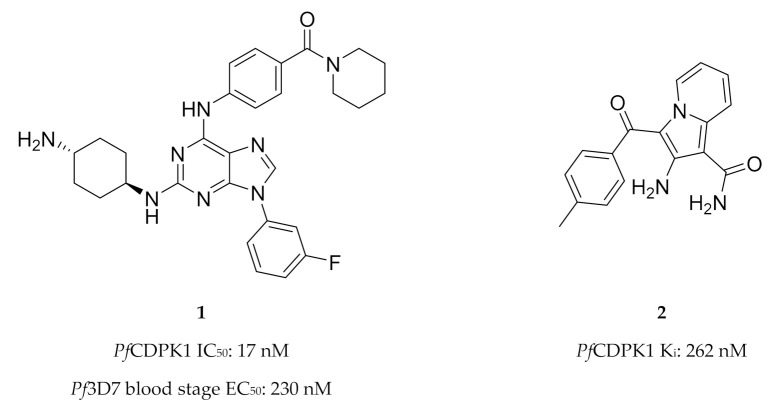

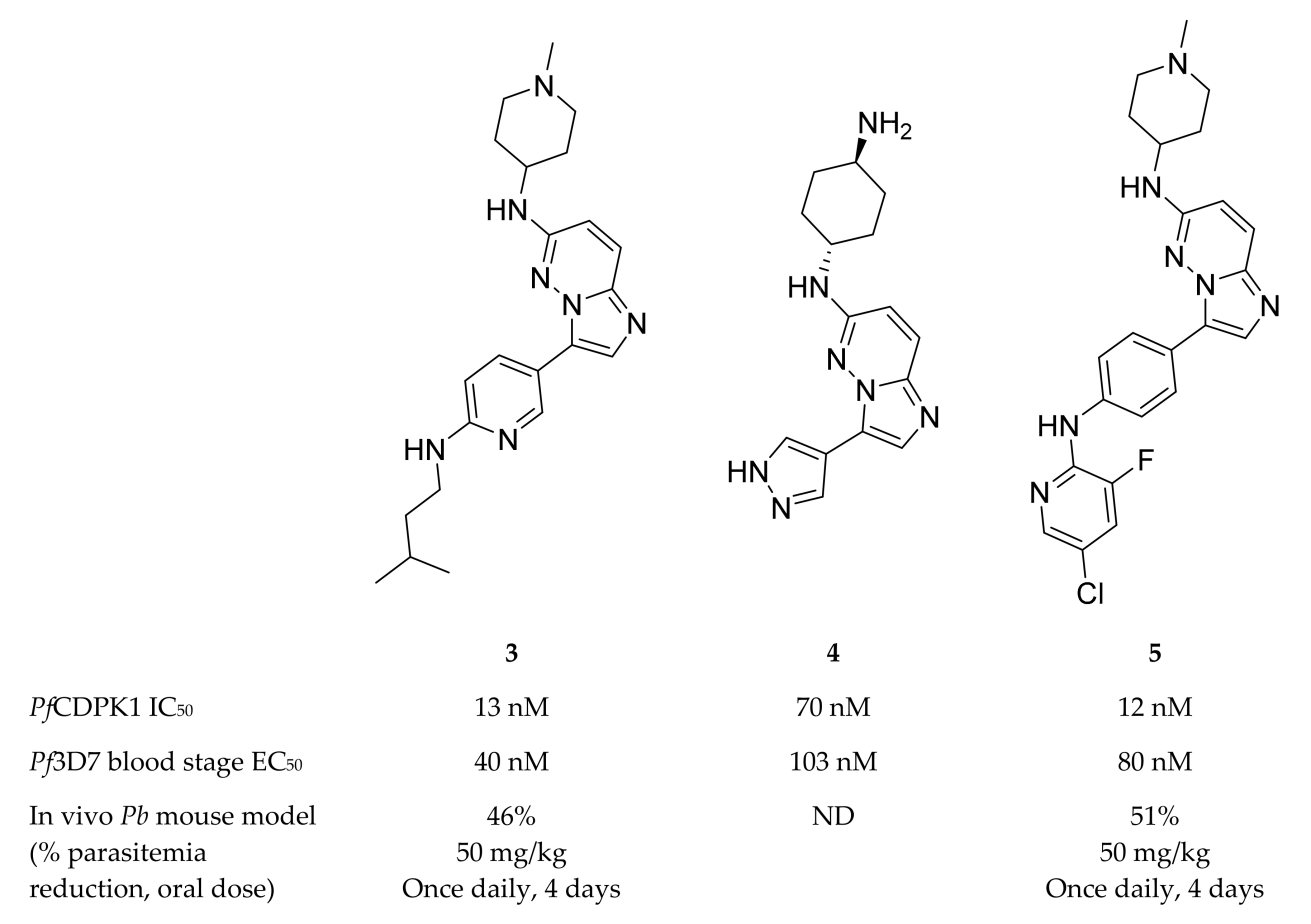
The different scaffolds identified as inhibitors of *Pf*CDPK1. IC_50_: half-maximal inhibitory concentration; *Pf*3D7: *Plasmodium falciparum* 3D7 strain; EC_50_: half-maximal effective concentration; K_i_: inhibitory constant; *Pb*: *Plasmodium berghei*; ND: not determined.

High-throughput screening campaigns have identified various scaffolds as *Pf*CDPK1 inhibitors (Figure 1), including 2,3,9-trisubstituted purines (**1**) [19], indolizines (**2**) [38] and imidazopyridazines (**3**–**5**) [23,38,39,40,41,42]. Of all these scaffolds, imidazopyridazines have been studied more intently.

**Figure 2:**
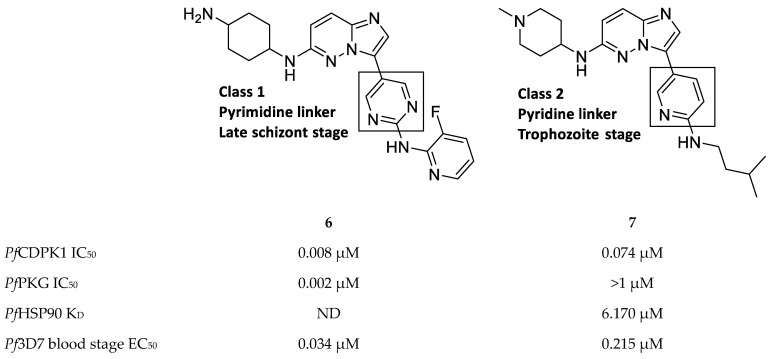
Class 1 and 2 imidazopyridazine-based *Pf*CDPK1 inhibitors. K_D_: equilibrium dissociation constant. ND: not determined.

Imidazopyridazines are generally potent *Pf*CDPK1 inhibitors, with some compounds demonstrating low micromolar to submicromolar activity against recombinant *Pf*CDPK1 and *P. falciparum* erythrocytic parasites [23,38,39,40,41,42]. However, discrepancies between the enzymatic and whole-cell activities are generally reported for these compounds, which may be due to off-target activity or permeability issues [38,39]. Considerable effort was made to improve the selectivity and ADME (absorption, distribution, metabolism, excretion) profiles of imidazopyridazines, which resulted in compounds with well-balanced *Pf*CDPK1 potency, permeability and in vitro activity against *P. falciparum* erythrocytic stage parasites [40,41,42]. Despite these efforts, studies still reported only modest in vivo activity in a *P. berghei* mouse model [39,40,41]. Further exploration of the mechanism of action of imidazopyridazines demonstrated that these compounds could be grouped into two classes based on the type of aromatic linker between the core and the R2 substituent (Figure 2) [23]. Class 1 compounds (**6**) had a pyrimidine linker and inhibited *P. falciparum* parasite growth at the late schizont stage, while class 2 compounds (**7**) had a non-pyrimidine linker and inhibited the trophozoite stage of *P. falciparum*. Two additional parasitic targets were also identified for imidazopyridazines: class 1 compounds inhibited *Pf*PKG and class 2 compounds inhibited *Pf*HSP90 (a chaperone protein of *P. falciparum*). These results suggest that the activity of imidazopyridazines against erythrocytic *P. falciparum* parasites is primarily due to inhibition of *Pf*PKG and *Pf*HSP90, rather than *Pf*CDPK1 inhibition.

More recently, Flaherty and co-workers [43] designed a hydrocarbon constrained peptide that mimics the C-terminal helical region of the *Pf*CDPK1 junction domain (J-domain). The autoinhibitory J-domain is located between the catalytic domain and the calmodulin-like domain and blocks the active site by acting as a pseudosubstrate when the kinase is in its inactive state. By mimicking the activity of the J-domain, the constrained peptide inhibits *Pf*CDPK1 by locking the kinase in its inactive state. Uptake of the constrained peptide by *P. falciparum-*infected erythrocytes was highly stage-specific, as late-stage schizont erythrocytes demonstrated increased uptake relative to ring-stage and early trophozoite erythrocytes. The constrained peptide inhibited recombinant *Pf*CDPK1 in the low micromolar range (IC_50_: 3.5 µM) and caused a significant decrease in parasitemia at concentrations of ≥10 µM.

**Figure 3:**
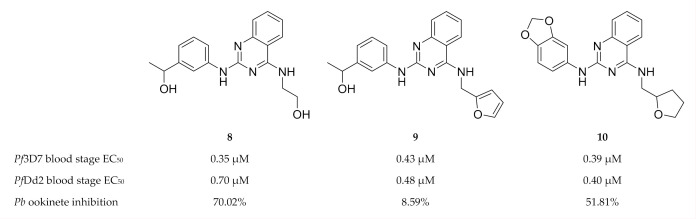
Quinazoline derivatives as inhibitors of multiple plasmodial kinases.

Lima and co-workers [44] designed and developed shape-based and machine learning models of *Pf*CDPK1, *Pf*CDPK4 and *Pf*PK6. These models were used for virtual screening of drug-like molecules to identify potent inhibitors with activity against multiple *P. falciparum* kinases. The computational hits were then evaluated in vitro against drug-sensitive (3D7) and multidrug-resistant (Dd2) *P. falciparum* erythrocytic parasites. Quinazoline derivatives (compounds **8**–**10**, Figure 3) inhibited the growth of both drug-sensitive and multidrug-resistant *P. falciparum* strains in the nanomolar range. Compounds **8** and **10** also demonstrated good in vivo inhibition of *P. berghei* ookinete formation at a concentration of 10 µM. Molecular docking studies indicated that compound **8** was able to interact with *Pf*CDPK1, *Pf*CDPK4 and *Pf*PK6, thus highlighting its potential as a multi-kinase inhibitor.

**Figure 4:**
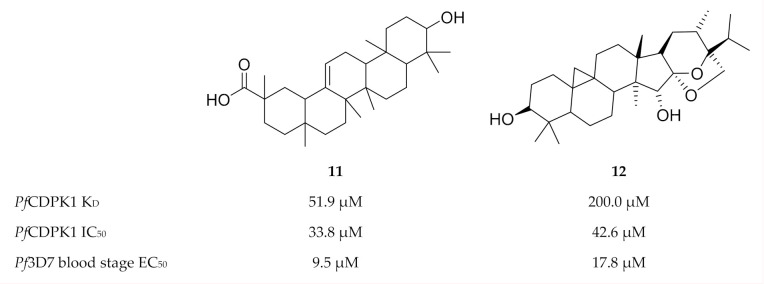
Structures and biological data of compounds **11** and **12**.

Another virtual screening campaign against a *Pf*CDPK1 homology model (*Pb*CDPK1 crystal structure, PBD ID: 3Q5I), identified 18 compounds from the MyriaScreen Diversity Library II that complement the *Pf*CDPK1 ATP-binding site [45]. Two of these compounds, **11** (ST092793) and **12** (S344699) (Figure 4), significantly inhibited recombinant *Pf*CDPK1 and demonstrated in vitro activity against *P. falciparum* erythrocytic parasites. Interestingly, isothermal titration calorimetry and fluorescence spectroscopy showed that **11** preferentially binds to the inactive conformation of *Pf*CDPK1, thereby locking the enzyme in this state throughout the erythrocytic stage.

Overall, the results from these studies suggest that *Pf*CDPK1 may not be the most suitable target for *P. falciparum* blood-stage infections. It seems as though the pathways and/or enzymes that are able to compensate for the loss of *Pf*CDPK1 activity [21,23,24] greatly affect the potency of *Pf*CDPK1 inhibitors in vivo. Greater success may be achieved if future drug development focusses on *Pf*CDPK1 as a potential transmission-blocking target.

#### 2.1.2. *Pf*CDPK4

As *Pf*CDPK4 is essential for sexual stage development of *P. falciparum*, it is a promising drug target for developing new transmission-blocking antimalarial drugs. The scaffolds explored thus far for *Pf*CDPK4 inhibition include phenothiazines, pyrazolopyrimidines, imidazopyrazines and 5-aminopyrazole-4-carboxamide derivatives [46,47,48,49,50].

**Figure 5:**
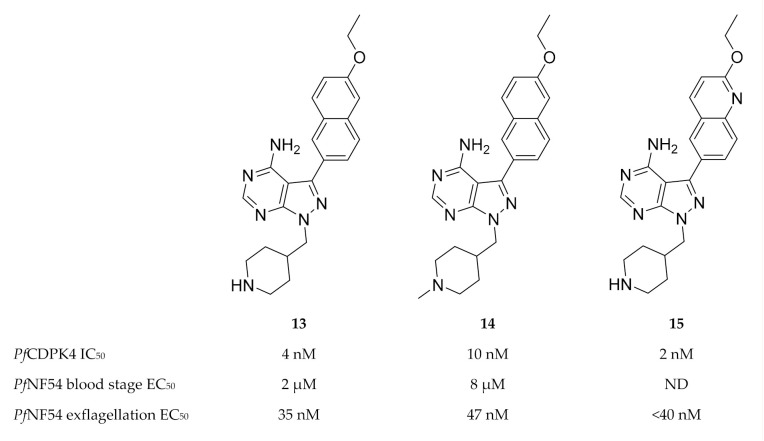
Pyrazolopyrimidine derivatives as inhibitors of *Pf*CDPK4. *Pf*NF54: *Plasmodium falciparum* NF54 strain.

Based on pyrazolopyrimidine BKIs designed for *Toxoplasma gondii* CDPK1 (*Tg*CDPK1) and *Cryptosporidium parvum* CDPK1 (*Cp*CDPK1), a series of pyrazolopyrimidine derivatives (e.g., compounds **13**–**15**, Figure 5) with potent activity against *Pf*CDPK4 were designed [49,50]. 

Minimal off-target activity was observed for some of these compounds when tested against human Src and Abl tyrosine kinases, which both have one of the smallest gatekeeper residues (threonine) in the human kinome [49,50]. However, when screened against human non-kinase targets, compound **14** also inhibited the human ether-a-go-go related gene potassium channel (hERG) which is critical for cardiac repolarisation [51]. Pyrazolopyrimidine compounds have been shown to block exflagellation of male gametocytes in *P. falciparum* parasites [49,50] and in *P. berghei*-infected mice [49] within the nanomolar range. When *Anopheles stephensi* mosquitoes were allowed to feed on *P. berghei*-infected mice treated with compound **13** (10 mg/kg, intraperitoneally), oocyst formation was blocked in the mosquito midgut. Similarly, infective sporozoite formation was inhibited in *Anopheles stephensi* mosquitoes that fed on *Pf*NF54-infected human blood containing 3 µM of compound **13** [49]. *P. falciparum* parasites expressing *Pf*CDPK4 with a mutated gatekeeper (small serine residue changed to a large methionine residue, S147M), were insensitive to pyrazolopyrimidine-based compounds and demonstrated normal exflagellation, which confirms *Pf*CDPK4 to be the target of these inhibitors [48,49]. Substituting the pyrazolopyrimidine scaffold with an imidazopyrazine core generally resulted in similar *Pf*CDPK4 selectivity and potency [50]. In silico studies revealed the structure-activity relationships of these pyrazolopyrimidine and imidazopyrazine compounds with the *Pf*CDPK4 target [52]. 

**Figure 6:**
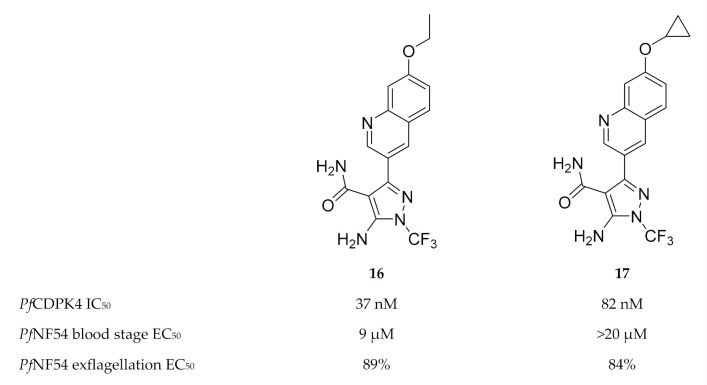
5-Aminopyrazole-4-carboxamide derivatives as inhibitors of *Pf*CDPK4.

Another scaffold used for *Tg*CDPK1 inhibitor development [53], 5-aminopyrazole-4-carboxamide, was also shown to potently inhibit *Pf*CDPK4 in the nanomolar range [47]. The most active 5-aminopyrazole-4-carboxamide derivatives (compounds **16** and **17**, Figure 6) demonstrated potent inhibition of *P. falciparum* male gametocyte exflagellation at a concentration of 0.1 µM. The in vitro inhibition was much higher than the enzymatic assay predicted, which may indicate multiple targets for these compounds. In terms of selectivity over human kinases, these inhibitors demonstrated high selectivity over Src kinase and hERG.

**Figure 7:**
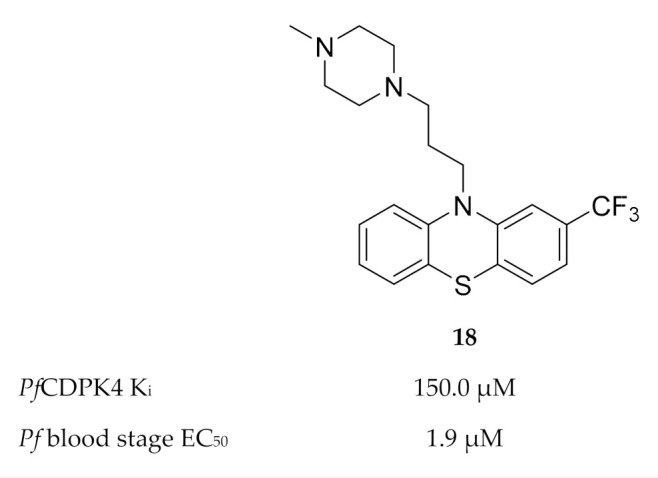
Structure and biological data of trifluoperazine (TFP).

Members of the phenothiazine class were identified as possible non-ATP-competitive inhibitors of *Pf*CDPK4 [46]. Trifluoperazine (TFP) (**18**, Figure 7) was the most active of this class, with a binding affinity (K_d_) of 134.5 µM and K_i_ value of 150 µM for *Pf*CDPK4. The discrepancy between the enzymatic activity of TFP and the reported in vitro activity (EC_50_: 1.9 µM) against *P. falciparum* indicates that this compound modulates multiple targets. 

Homology modelling indicated that TFP possibly binds to the calmodulin-like domain of *Pf*CDPK4 which prevents repositioning of the autoinhibitory J-domain upon binding of Ca^2+^, thereby locking the kinase in its inactive state.

#### 2.1.3. *Pf*CDPK5

**Figure 8:**
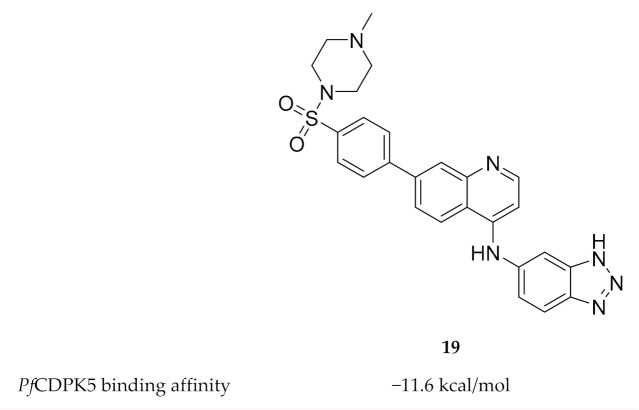
Structure and biological data of MMV687246.

To date, only one study has been published on inhibitor development for *Pf*CDPK5. Rout and Mahapatra predicted the three-dimensional structure of *Pf*CDPK5 through homology modelling using *P. berghei* CDPK1 as a template [54]. Possible inhibitors of *Pf*CDPK5 were then identified through virtual screening of five different sets of compounds with known antimalarial activity. MMV687246 (**19**, Figure 8), from the Malaria box assembled by The Medicines for Malaria Venture, demonstrated the highest binding affinity for *Pf*CDPK5 and was suggested as a possible lead for future experimental validation and inhibitor design.

## 3. AGC Group

Three of the five malarial kinases that cluster within this group, namely adenosine monophosphate (cAMP)-dependent protein kinase A (PKA), cyclic guanosine monophosphate (cGMP)-dependent protein kinase G (PKG) and protein kinase B (PKB) have been characterised [13]. cAMP-, cGMP- and calcium-mediated signalling pathways are closely linked within the malaria parasite. In merozoites, a rise in cytosolic cAMP levels leads to activation of PKA and an increase in cytosolic calcium levels via induction of the Epac (exchange protein directly activated by cAMP) pathway [55]. When activated by cGMP, PKG regulates phosphoinositide metabolism which produces inositol (1,4,5)-triphosphate (IP_3_), a messenger molecule that signals the release of intracellular calcium [56]. The release of calcium in turn activates stage-specific effector pathways, including CDPK signalling [29,56]. In contrast to the other two kinases, PKB is activated by calmodulin in a calcium-dependent manner [57]. Phospholipase C has been identified as the upstream regulator responsible for releasing the calcium required for PKB activation [57].

During the asexual parasite stages, *Pf*PKA, *Pf*PKG and *Pf*PKB regulate different factors required for parasite invasion and egress. *Pf*PKA phosphorylates the *P. falciparum* apical membrane antigen 1 (*Pf*AMA1) which is critical for tight junction formation between the parasite and the host cell during erythrocyte invasion [58,59,60]. *Pf*PKA has also been implicated in regulation of parasite motility [61], microneme secretion [55], anion channel conductance at the erythrocytic plasma membrane [62] and the cell cycle of the intraerythrocytic parasite [63]. However, Patel and co-workers [60] reported that events prior to invasion, such as egress, rise in cytosolic calcium levels and microneme secretion, can all occur in the absence of cAMP and *Pf*PKA. Apart from merozoite invasion, they also did not observe any other critical role for cAMP and *Pf*PKA in the erythrocytic life cycle. *Pf*PKG controls invasion and egress of sporozoites (liver-stage parasites) [29,64] and merozoites (blood-stage parasites) [65,66,67] by regulating parasite motility and microneme secretion. *Pf*PKB also plays a role in merozoite invasion of erythrocytes by regulating parasite motility [68]. During the sexual parasite stages, *Pf*PKG regulates gametogenesis, which involves male gamete exflagellation and rounding up of female gametes, and ookinete motility required for mosquito midgut invasion [56,69,70]. Both *Pf*PKA and *Pf*PKG have been validated as essential kinases, *Pf*PKA being essential for blood-stage parasites [58,60] and *Pf*PKG being essential for multiple life cycle stages [67,69,71]. *Pf*PKB is regarded as likely essential to blood-stage parasite survival [22].

### 3.1. Inhibitor Development for the AGC Group 

#### 3.1.1. *Pf*PKG

In terms of inhibitor development, *Pf*PKG is one of the plasmodial kinases that has been studied extensively thus far. It is regarded as a very attractive antimalarial drug target as its inhibition offers simultaneous prophylactic, curative and transmission-blocking potential [72]. As *Pf*PKG is an essential enzyme for multiple life cycle stages, there is a relatively low risk of the parasite developing high-grade resistance to *Pf*PKG-selective inhibitors [72]. The PKG enzyme is also highly conserved in all human malaria species, with an overall sequence identity of 90–92% and identical catalytic site and gatekeeper residues [73]. Thus, PKG inhibitors have the potential to be active against multiple malaria species. Although human PKG orthologues (cytosolic PKG-Iα and PKG-Iβ, membrane-associated PKG-II) exist, selective inhibitor development is still possible as there is significant structural divergence between plasmodial and mammalian PKGs [74].

**Figure 9:**
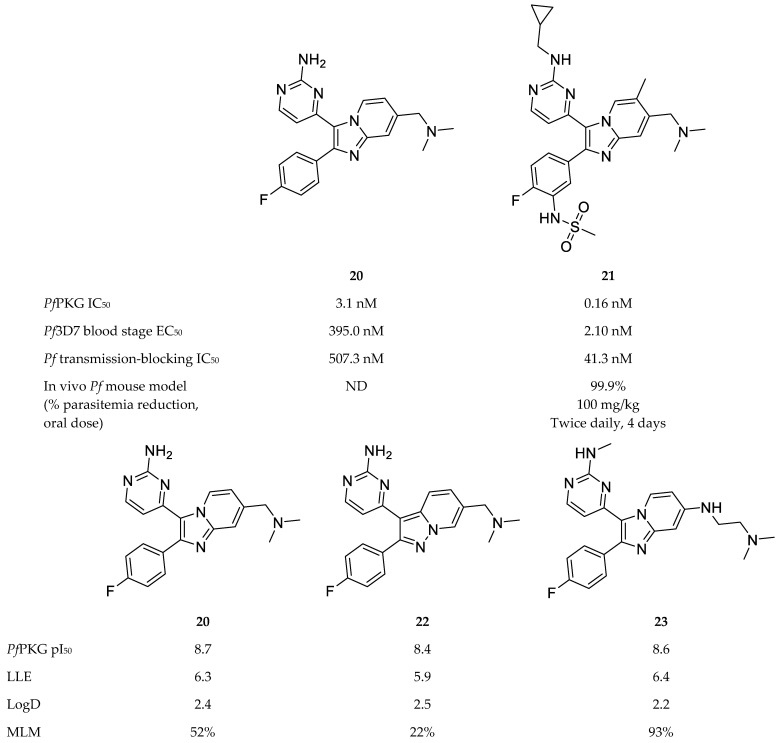
Imidazopyridine-based compounds as inhibitors of *Pf*PKG. pI_50_: negative log of the IC_50_ value in molar; LLE: lipophilic ligand efficiency; LogD: distribution coefficient; MLM: % remaining after 30 min incubation with mouse live microsomes.

A variety of scaffolds have been explored for *Pf*PKG inhibitor development, with the imidazopyridines [71,75,76] and thiazoles [73,77,78] being the most advanced inhibitors of *Pf*PKG. Compound **20** (Figure 9), a PKG inhibitor of *Eimeria tenella* [79], was used as a lead compound to develop potent imidazopyridine inhibitors of *Pf*PKG [71,75,76]. Compound **21** (Figure 9) was the most potent of a series of imidazopyridines synthesised by Baker and co-workers [71], with an IC_50_ value of 0.16 nM against *Pf*PKG and an EC_50_ value of 2.1 nM against *P. falciparum* blood-stage parasites. Compound **21** demonstrated no toxicity in vitro or in vivo, high selectivity over human kinases and moderate metabolic stability in vitro. Oral administration of compound **21** to *P. falciparum*-infected mice (twice-daily dose of 100 mg/kg) over a period of four days reduced parasitemia below detectable levels. Apart from blood-stage efficacy, compound **21** also inhibited transmission of mature *P. falciparum* gametocytes to *Anopheles stephensi* mosquitoes (IC_50_: 41.3 nM).

Subsequent studies focussed on improving the ADME properties of this chemical class while retaining potent inhibitory activity. By means of structure-activity relationship and modelling studies, Large and co-workers [75,76] systematically varied the substituents of compound **20** and explored other bicyclic cores. Compounds **22** [75] and **23** [76] (Figure 9) retained the potent *Pf*PKG and in vitro antimalarial activity of compound **20**. Compound **22** also retained the LogD and lipophilic ligand efficiency of compound **20**, and showed improved permeability, but demonstrated poor stability in mouse liver microsomes [75]. Compound **23** demonstrated an excellent balance of activity and physicochemical properties. In addition to good LogD and LLE, compound **23** showed improved microsomal stability and excellent selectivity over human kinases, including the two human PKG orthologues (PKG1α and PKG1β) [76].

**Figure 10:**
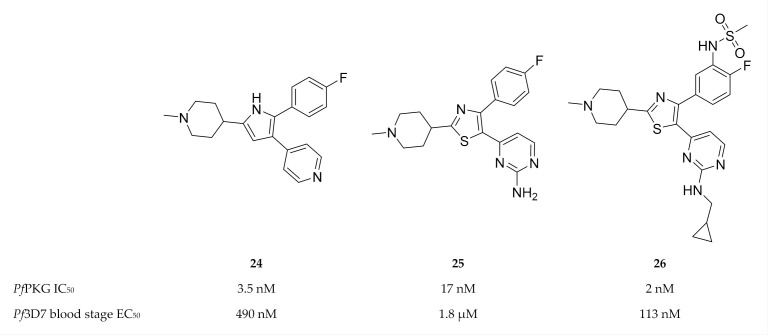
2,3-Diaryl-pyrrole and thiazole compounds as inhibitors of *Pf*PKG.

A 2,3-diaryl-pyrrole inhibitor of PKG developed for *Eimeria tenella* (compound **24**, Figure 10) [80] was shown to be a potent inhibitor of *Pf*PKG (IC_50_: 3.5 nM) [81]. However, compound **24** only demonstrated modest in vitro activity against *P. falciparum* and failed to reduce parasitemia in a *P. berghei* mouse model [81]. A scaffold-hopping approach performed on compound **24** lead to the identification of thiazoles (compound **25**) as *Pf*PKG inhibitors [78]. Substitution of the thiazole scaffold was optimised to improve the enzymatic and in vitro activity of compound **25**, which lead to compound **26** (IC_50_: 2 nM, EC_50_: 113 nM) [78]. Compound **26** demonstrated excellent selectivity over human kinases, good permeability and metabolic stability in mouse and human liver microsomes.

**Figure 11:**
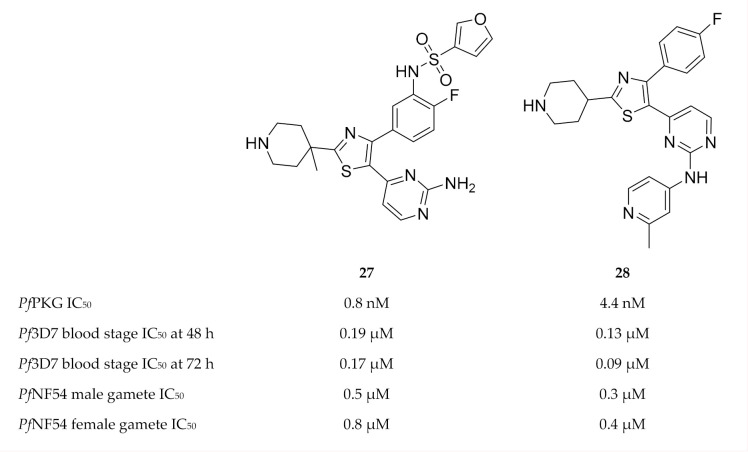
Thiazole derivatives as inhibitors of multiple plasmodial kinases.

During a high-throughput screening campaign, Penzo and co-workers [73] also identified several thiazole derivatives, such as compound **27** (Figure 11), with nanomolar potencies against *Pf*PKG [73]. The in vitro activity of the most potent compounds was studied at 48 and 72 h in wild-type and transgenic (*Pf*PKG gatekeeper mutant, T618Q) *P. falciparum* blood-stage parasites, and the EC_50_ values against the two strains were very similar—indicating that the thiazole derivatives also inhibit targets other than *Pf*PKG. Additional targets identified for compound **27** included CDPK1, CDPK4, CDK-related kinase (Pfcrk-5), NIMA-related kinase (*Pf*nek-1), CK1 and an unnamed putative protein kinase (Pf3D7_0926100). Compound **27** also demonstrated good solubility, no toxicity against HepG2 cells, and selectivity over the human PKG orthologue (PKGIα), human lymphocyte-specific protein tyrosine kinase (LCK) and human Aurora B kinase. Besides blood-stage activity, compound **27** also had potent activity against male and female gametes.

Matralis and co-workers [77] specifically focussed on developing a series of thiazole derivatives with a fast-killing profile similar to that of artemisinins. Compound **28** (Figure 11) was the most potent in this series, with in vitro nanomolar activity against *Pf*PKG, *P. falciparum* blood-stage parasites and gametocytes. It also possessed good physicochemical properties. Despite good selectivity over human enzymes, ion channels and receptors, this compound showed activity towards hERG. Parasite reduction ratio studies demonstrated that compound **28** had fast-killing properties similar to those of artesunate. CDPK1, CDPK4 and serine/arginine protein kinase 2 (SRPK2, also known as CLK2) were identified as additional targets of compound **28**. The fast-killing activity of compound **28** was mainly attributed to SRPK2 inhibition. This study demonstrates the potential of simultaneously targeting *Pf*PKG and SRPK2 to develop fast-killing drugs with curative and transmission-blocking activity.

**Figure 12:**
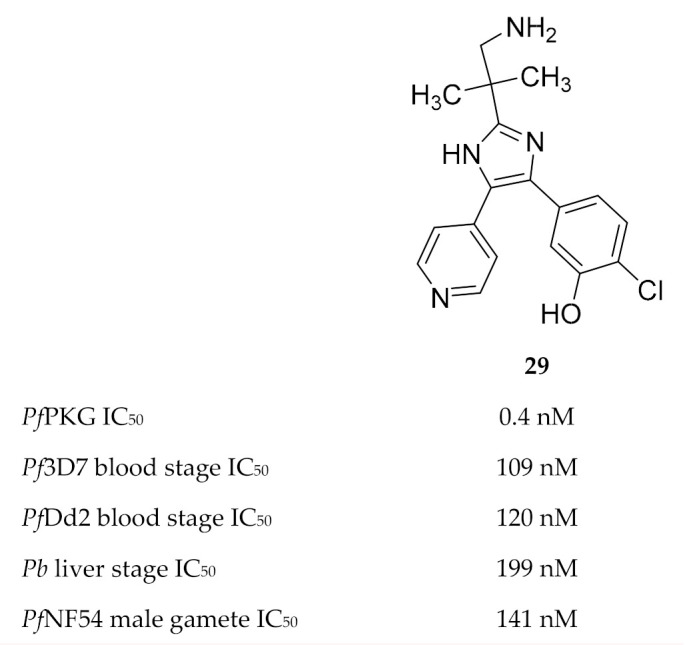
Structure and biological data of MMV030084.

Vanaerschot and co-workers [72] identified *Pf*PKG as the primary target of the Medicines for Malaria Venture compound MMV030084 (**29**, Figure 12). MMV030084 had an IC_50_ value of 0.4 nM against recombinant *Pf*PKG, and docking studies using the *Pf*PKG crystal structure (PDB ID: 5DYK) showed a strong interaction between MMV030084 and the ATP-binding site. When evaluated against the different life cycle stages of the parasite, MMV030084 inhibited sporozoite invasion of hepatocytes, merozoite egress from mature schizonts, and male gamete exflagellation. MMV030084 inhibited liver cell invasion by *P. berghei* parasites (IC_50_: 199 nM) with minimal toxicity against the host cells (CC_50_: 41.5 µM). The development of *P. falciparum* blood-stage parasites was halted at the schizont stage when treated with MMV030084 (drug-sensitive strain IC_50_: 109 nM; multidrug-resistant strain IC_50_: 120 nM). Male gamete exflagellation of the *P. falciparum* NF54 strain was inhibited by MMV030084 with an IC_50_ value of 141 nM. In vitro MMV030084 resistance selection studies identified *P. falciparum* tyrosine kinase-like 3 (*Pf*TKL3) as an MMV030084-resistance mediator, which allows merozoite egress from erythrocytes when mutated. No mutation was identified for *Pf*PKG itself.

**Figure 13:**
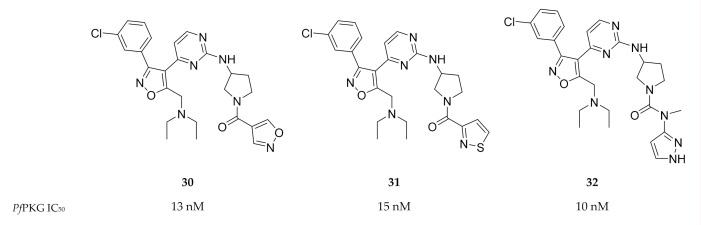
Isoxazole-based inhibitors of *Pf*PKG.

A screening campaign by Mahmood and co-workers [82] identified isoxazole-based inhibitors with *Pf*PKG activity and selectivity over human PKG. Optimisation of this scaffold led to compounds **30**–**32** (Figure 13) with IC_50_ values <20 nM against *Pf*PKG. Further evaluation of the physicochemical properties and in vitro activity of these compounds against the whole-cell parasite has not yet been reported.

#### 3.1.2. *Pf*PKA and *Pf*PKB

**Figure 14:**
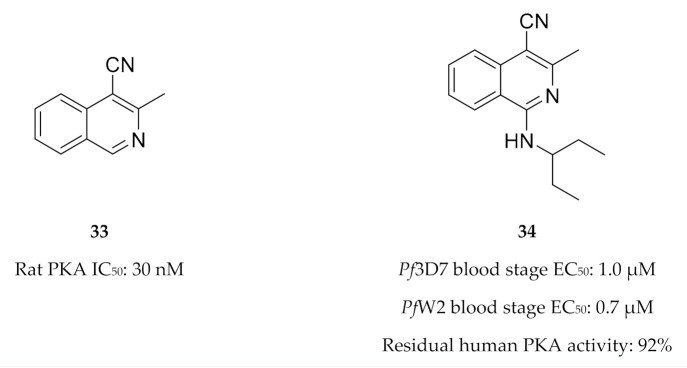
3-Methylisoquinoline-4-carbonitrile derivatives and their biological data.

Drug discovery efforts targeting *Pf*PKA and *Pf*PKB are limited. Buskes and co-workers [83] attempted to develop *Pf*PKA inhibitors using the commercially available PKA inhibitor 3-methylisoquinoline-4-carbonitrile (**33**, Figure 14) as a starting point. They studied the interactions of compound **33** and a series of substituted isoquinolines (e.g., **34**, Figure 14) with a *Pf*PKA homology model. In vitro evaluation of this series demonstrated low micromolar activity against drug-sensitive (3D7) and -resistant (W2) *P. falciparum* strains. However, biochemical evaluation of this series showed minimal activity against PKA [84]. It was suggested that these compounds likely inhibit another kinase that is involved in parasitic cytokinesis and erythrocyte invasion [84].

**Figure 15:**
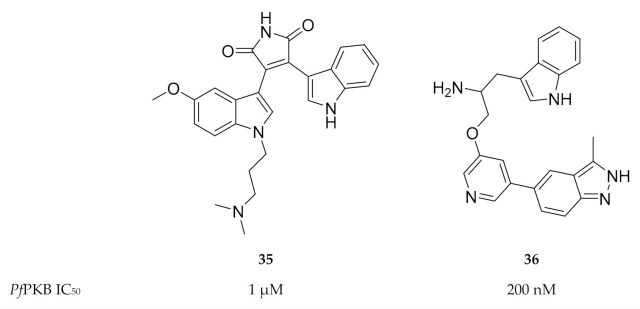
Structures and biological data of Go 6983 and A443654.

As *Pf*PKB shares high sequence homology with the catalytic sites of mammalian PKB and protein kinase C (PKC), established PKB and PKC inhibitors were tested for activity against *Pf*PKB. Go 6983 (**35**, Figure 15), a PKC inhibitor, was shown to inhibit *Pf*PKB (IC_50_: ±1 µM) and significantly reduce *P. falciparum* parasite growth at the late schizont stage [85]. The mammalian PKB inhibitor A443654 (**36**, Figure 15) inhibited *Pf*PKB with an IC_50_ value of 200 nM [57]. Incubation of *P. falciparum* schizonts with A443654 did not affect the morphology or the number of schizonts but dramatically reduced the number of ring-stage parasites formed after invasion. This indicates that A443654 blocks invasion, which corroborates the function of *Pf*PKB in erythrocytic invasion. A peptide inhibitor that corresponds to the pseudosubstrate motif of the *Pf*PKB N-terminal effectively inhibited *Pf*PKB activity and also reduced the formation of *P. falciparum* ring-stage parasites [57].

Crystal structures of *Pf*PKA’s regulatory subunit (PDB ID: 5K8S, 5KBF and 5T3N) [86] and methods for expression and purification of active recombinant *Pf*PKAr (regulatory subunit) [86] and *Pf*PKAc (catalytic subunit) [87] are available for future drug development efforts for this target. The crystal structure of *Pf*PKB is not yet available; however, the full-length *Pf*PKB gene has been successfully expressed and purified as an active recombinant protein [57,68,85].

## 4. CMGC Group

The CMGC kinase family consists of cyclin-dependent kinases (CDKs), mitogen-activated protein kinases (MAPKs), glycogen synthase kinase-3 (GSK-3) and CDK-like kinases (CLKs).

### 4.1. Cyclin-Dependent Kinases (CDKs)

Eukaryotic cyclins and CDKs are essential regulators of the cell cycle [88]. Several CDK homologues have been identified in the *P. falciparum* parasite, namely protein kinase 5 (*Pf*PK5), protein kinase 6 (*Pf*PK6), MO15-related kinase (*Pf*mrk) and the CDK-related kinases (*Pf*crk-1, *Pf*crk-3, *Pf*crk-4, *Pf*crk-5) [88,89].

*Pf*PK5 is most related to mammalian CDK1 and CDK5 [90] and demonstrates sensitivity to mammalian CDK1/CDK2 inhibitors [91]. It has been proposed that *Pf*PK5 is likely involved in the regulation of DNA replication (S-phase of the cell cycle) during erythrocytic schizogony [90,92,93]. *Pf*mrk, a putative homologue of mammalian CDK7, is predominantly expressed in gametocytes and to a lesser extent in the asexual stages (trophozoites and schizonts) [94]. *Pf*mrk is localised in the nucleus of the parasite and is presumably involved in the regulation of DNA replication [95]. *Pf*crk-1 and *Pf*crk-3 display maximal homology to mammalian CDKs involved in transcriptional control [13,96]. *Pf*crk-1 is mainly expressed in gametocytes [97]; however, the *P. berghei* orthologue (*Pb*crk-1) was found to be essential for erythrocytic schizogony [98]. *Pf*crk-3 has been demonstrated to be essential to erythrocytic parasites and presumably regulates gene expression via interaction with chromatin modification enzymes [96]. *Pf*crk-4 is another essential enzyme for asexual proliferation and also plays a critical role in ookinete formation and early oocyst development [22,99]. Two atypical CDKs, namely *Pf*PK6 and *Pf*crk-5, demonstrate both CDK and mitogen-activated protein kinase (MAPK) homology [100,101]. Nuclear and cytoplasmic localisation have been reported for *Pf*PK6 in trophozoite and schizont parasites [100]. Unlike *Pf*PK5 and *Pf*mrk which are activated by various cyclins in vitro [102,103], *Pf*PK6 appears to be a cyclin-independent kinase [100]. *Pf*crk-5 is a cyclin-dependent enzyme localised in the nuclear periphery [101]. Although parasites lacking *Pf*crk-5 are viable, they display decreased erythrocytic proliferation due to a lower number of merozoites released per schizont [101].

#### 4.1.1. CDK Inhibitor Development

##### *Pf*PK5

**Figure 16:**
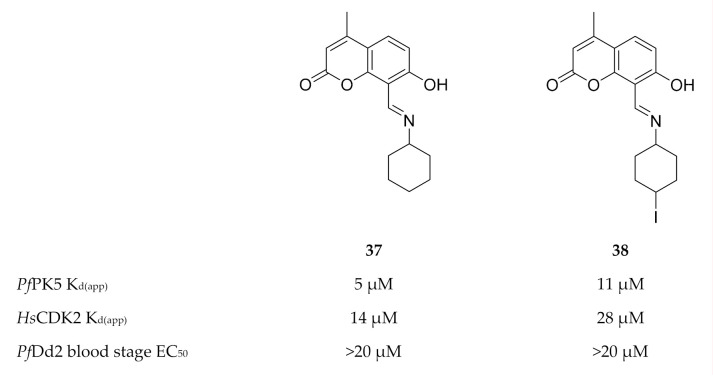
4-Methylumbelliferone inhibitors of *Pf*PK5 and their biological data. K_D(app)_: apparent dissociation constant.

Developing *Pf*PK5-selective inhibitors is quite challenging due to the high degree of homology between *Pf*PK5 and the human CDKs. Eubanks and co-workers [104] demonstrated that a target-based screening approach was more effective to identify *Pf*PK5-selective inhibitors than chemically modifying existing CDK inhibitors. The 4-methylumbelliferone analogues, compounds **37** and **38** (Figure 16), demonstrated a 2-fold binding affinity for *Pf*PK5 over human CDK2 (*Hs*CDK2). No significant toxicity was observed against human hepatoma cell lines (HuH7 and HepG2) for either compound. However, both compounds failed to inhibit drug-resistant *P. falciparum* (Dd2 strain) asexual parasite growth in vitro, most likely due to poor physicochemical properties.

##### *Pf*mrk

Scaffolds studied for *Pf*mrk inhibition include quinolinones [105], oxindoles [106], chalcones [107], flavonoids [108] and sulfonamide-based compounds [109,110,111].

**Figure 17:**
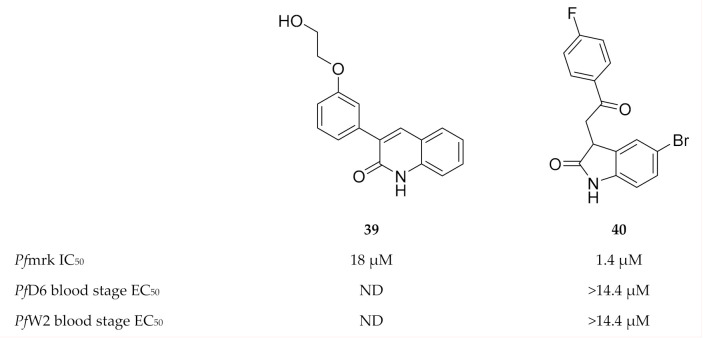
Quinolinone and oxindole derivatives as inhibitors of *Pf*mrk.

A series of quinolinones (e.g., compound **39**, Figure 17) demonstrated *Pf*mrk activity with IC_50_ values ranging from 18 to 539 µM [105]. Neither antimalarial evaluation against whole-cell parasites nor molecular docking has been performed for this chemical class. Based on the structure of commercially available indirubin-3′-monoxime, a moderate inhibitor of *Pf*mrk and in vitro *P. falciparum* parasite growth, oxindole-based compounds were explored as inhibitors of *Pf*mrk [106]. Several oxindoles selectively inhibited *Pf*mrk in the low micromolar range, with the most potent being compound **40** (Figure 17). None of the oxindoles demonstrated any significant in vitro activity against whole-cell parasites. Homology modelling showed that these compounds had the same orientation in the active site of *Pf*mrk as in human CDK2, but with additional contact points which might be responsible for the *Pf*mrk specificity. 

**Figure 18:**
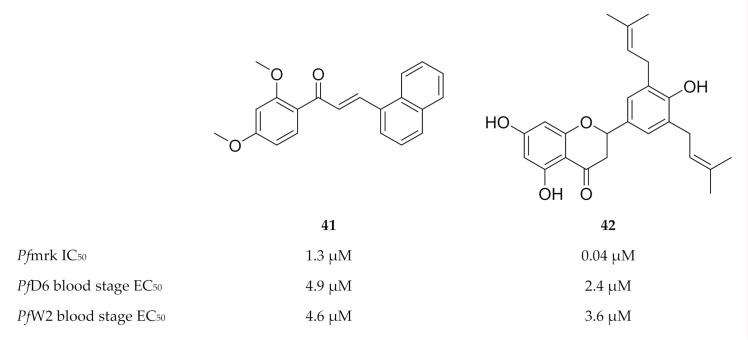
Structures and biological data of chalcone and flavonoid-based inhibitors of *Pf*mrk.

The chalcone and sulfonamide-based scaffolds were identified by means of a three-dimensional structure-activity relationship (3D-QSAR) pharmacophore model [109,112]. Compound **41** (Figure 18) was the most active compound of a series of chalcones tested against *Pf*mrk (IC_50_: 1.3 µM) [107]. However, a weak correlation was observed between the *Pf*mrk activity and the in vitro activity against drug-sensitive (D6) and -resistant (W2) *P. falciparum* strains. As several mechanisms of action have been reported for the antimalarial activity of chalcones [113,114,115], Geyer and co-workers [107] proposed that *Pf*mrk inhibition might be an additional mechanism demonstrated by some chalcones. Several flavonoids isolated from *Erythrina* sp., were evaluated for activity against *Pf*mrk [108]. The most potent flavonoid, Abyssinone V (**42**), had an IC_50_ value of 0.038 µM. Despite potent *Pf*mrk activity, the flavonoids demonstrated similar in vitro antimalarial activity to that of the chalcones, presumably due to the lower permeability of the flavonoids. 

**Figure 19:**
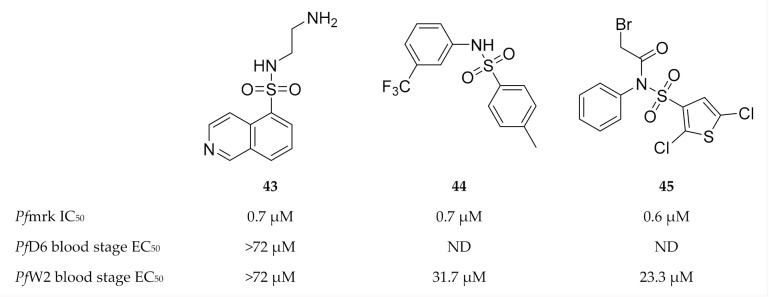
Sulfonamide-based inhibitors of *Pf*mrk.

Isoquinoline sulfonamides were generally weak inhibitors of *Pf*mrk, except compound **43** (Figure 19) which demonstrated an IC_50_ value of 0.7 µM [111]. However, compound **43** failed to inhibit drug-sensitive (D6) and -resistant (W2) *P. falciparum* parasite growth. Thiophene sulfonamides exemplified by **44** and **45** (Figure 19) were found to be potent inhibitors of *Pf*mrk with IC_50_ values in the submicromolar range [110]. However, all of these compounds, except compound **45**, are also potent inhibitors of human CDK7. Thiophene sulfonamides demonstrated minimal cytotoxicity and moderate in vitro activity against a multidrug-resistant *P. falciparum* (W2) strain. 

##### *Pf*PK6 and CDK-Related Kinases

To our knowledge, no medicinal chemistry campaigns have focussed on drug development for *Pf*PK6 and the CDK-related kinases (*Pf*crk-1 to -5). Homology models have been developed for *Pf*PK6 [116,117] and *Pf*crk-4 [99], which can be used for virtual screening of small-molecule inhibitors against these targets. Methods for expression and purification of active recombinant *Pf*PK6 [100], *Pf*crk-1 (kinase domain) [118], *Pf*crk-3 [96] and *Pf*crk-5 [101] are also available in the literature.

### 4.2. Mitogen-Activated Protein Kinases (MAPKs)

The *P. falciparum* kinome encodes two MAPK homologues, namely *Pf*map-1 and *Pf*map-2. Interestingly, *Pf*map-2 is essential for the asexual stages of *P. falciparum* parasites [119]; however, the *P. berghei* orthologue (*Pb*map-2) is only essential for male exflagellation in the mosquito midgut [120]. *Pf*map-1 also plays an important role during asexual development; however, parasites are able to compensate for loss of *Pf*map-1 activity by upregulating *Pf*map-2 [119].

Human p38 MAPK inhibitors have been shown to inhibit drug-sensitive (HB3) and -resistant *P. falciparum* strains in vitro; however, antiplasmodial activity has yet to be attributed to plasmodial MAPK inhibition [121]. 

### 4.3. Glycogen Synthase Kinase-3 (GSK-3)

*Pf*GSK-3 is one of three GSK3-related kinases identified in the *P. falciparum* parasite [13]. Although *Pf*GSK-3 is expressed throughout the erythrocytic stage, it is predominantly expressed during the early trophozoite stage [122]. After expression, *Pf*GSK-3 is rapidly transported to the cytoplasm of the erythrocyte where it appears to associate with membranous structures known as Maurer’s clefts [122]. The exact biological functions of *Pf*GSK-3 remain to be elucidated; however, it has been demonstrated to be essential for the survival of asexual erythrocytic parasites [22].

#### *Pf*GSK-3 Inhibitor Development

**Figure 20:**
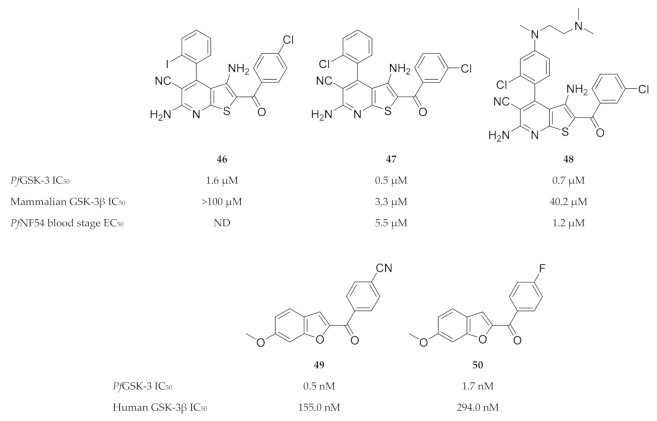
4-Phenylthieno[2,3-*b*]pyridine and benzofuran-based inhibitors of *Pf*GSK-3.

Despite the high degree of homology between *Pf*GSK-3 and mammalian GSK-3, significant structural differences exist that can be exploited for *Pf*GSK-3-selective inhibition [122,123,124,125]. Fugel and co-workers [123] designed a novel series of 4-phenylthieno [2,3-*b*]pyridine based on structures of hits (e.g., compound **46**, Figure 20) identified during a high-throughput screening campaign. Compounds from this series, such as compound **47** (Figure 20), selectively inhibited *Pf*GSK-3 (IC_50_: 0.48 µM) over human GSK-3 (GSK-3α IC_50_: >100 µM; GSK-3β IC_50_: 3.3 µM) and demonstrated broad selectivity when tested against two panels of mammalian kinases. Compound **47** also had in vitro activity against erythrocytic *P. falciparum* (NF54-Luc) parasites (EC_50_: 5.5 µM). Masch and co-workers [126] improved the solubility and antimalarial activity of the 4-phenylthieno[2,3-*b*]pyridine compounds by attaching an additional aliphatic polar side chain to the *para*-position of the 4-phenyl ring. Compared to compound **47**, compound **48** (Figure 20) exhibited 4.5-fold higher antiplasmodial activity against erythrocytic *P. falciparum* (NF54-Luc) parasites (EC_50_: 1.2 µM), as well as improved solubility (**47**: 4.8 µM; **48**: 1.5 µM). Recently, we identified a series of benzofuran-based compounds as potent and selective inhibitors of *Pf*GSK-3 ([127], submitted for publication). The most promising benzofurans, compounds **49** and **50** (Figure 20), inhibit *Pf*GSK-3 in nanomolar concentrations and demonstrate 316-fold and 175-fold selectivity for *Pf*GSK-3 over human GSK-3.

### 4.4. CDK-Like Protein Kinases (CLKs)

Four members of the CLK family have been identified in *P. falciparum*, *Pf*CLK-1 to -4 [13]. All four enzymes are essential to the asexual erythrocytic parasites [22,128] as they regulate mRNA splicing through phosphorylation of serine/arginine-rich (SR) proteins [129]. *Pf*CLK1 and *Pf*CLK2 exhibit homology to the yeast SR protein, Sky1p, and are expressed throughout the erythrocytic stage and in gametocytes [128]. Both kinases are localised within the nucleus of the parasite, with *Pf*CLK2 also present in the cytoplasm [128]. *Pf*CLK3 is a closely related homologue of human pre-mRNA processing factor 4B (PRP4 or PRPF4B) [130] which regulates mRNA splicing through phosphorylation of proteins associated with the spliceosome complex [131]. *Pf*CLK4, also known as SR protein-specific kinase 1 (SRPK1), is expressed in erythrocytic parasites and abundantly in gametocytes [132,133]. *Pf*CLK4 negatively regulates mRNA splicing by phosphorylating a putative plasmodial SR protein (*Pf*SR1) in vitro [132,134]. Both *Pf*CLK4 and *Pf*SR1 are localised inside the nucleus of ring and early trophozoite stage parasites. As erythrocytic development progresses, the two proteins are exported to the nuclear periphery (mature trophozoites) and finally to the cytoplasm (schizonts and gametocytes) [132,134].

#### CLK Inhibitor Development

**Figure 21:**
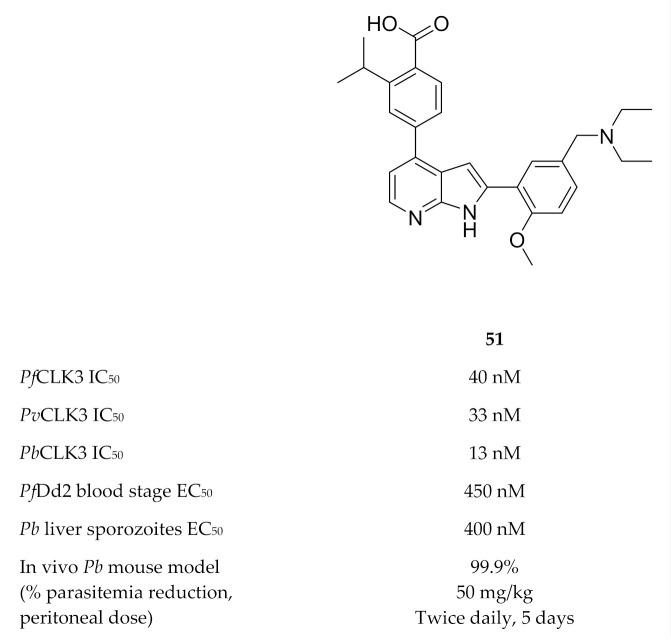
Structure and biological data of TCMDC-135051.

A high-throughput screening campaign identified TCMDC-135051 (**51**, Figure 21) as a highly potent and selective inhibitor of *Pf*CLK3 [135]. TCMDC-135051 demonstrated selectivity for *Pf*CLK3 over closely related human kinases (CLK2 and PRPF4B), the closest related plasmodial kinase (*Pf*CLK1) and two other plasmodial kinases (*Pf*PKG and *Pf*CDPK1). *Pf*CLK3 mutations were observed in *P. falciparum* parasites with reduced sensitivity to TCMDC-135051, which indicated that *Pf*CLK3 was the primary target of this compound. A recombinant *Pf*CLK3 variant with a G449P mutation and parasites expressing the G449P mutant *Pf*CLK3 both demonstrated reduced TCMDC-135051 sensitivity, confirming that the antimalarial activity of TCMDC-135051 was due to *Pf*CLK3 inhibition. TCMDC-135051 inhibition resulted in downregulation of 425 essential *P. falciparum* genes and upregulation of certain genes involved in RNA processing, which is consistent with the proposed mRNA splicing role of *Pf*CLK3. TCMDC-135051 was active against multiple stages of the parasite’s life cycle, including liver-stage sporozoites, blood-stage parasites, gametocyte development and subsequent transmission to the mosquito vector. TCMDC-135051 also demonstrated activity against CLK3 of *P. vivax* (*Pv*CLK3) and *P. berghei* (*Pb*CLK3), as well as in vitro activity against *P. knowlesi* and *P. berghei* blood-stage parasites. A dose-dependent reduction in parasitemia was observed when TCMDC-135051 was administered intraperitoneally to *P. berghei*-infected mice (twice daily, 5-day period), with the maximal dose (50 mg/kg) reducing parasitemia below detectable levels.

## 5. Casein Kinase 1 (CK1) Group

The *P. falciparum* kinome encodes a single CK1 enzyme (*Pf*CK1) that is expressed throughout the erythrocytic stages. *Pf*CK1 is essential for blood-stage parasite survival [22] and is likely involved in cellular processes such as mRNA splicing, protein trafficking and erythrocyte invasion [136,137]. To our knowledge, no medicinal chemistry programs have targeted this enzyme thus far.

## 6. NIMA- and Aurora-Related Kinases

Four Never in Mitosis, gene A (NIMA)-related kinases or NEKs (*Pf*nek-1 to -4) [12,13] and three Aurora-related kinases (*Pf*ark-1 to -3) [138] have been identified for *P. falciparum*. Both groups of kinases are involved in the regulation of the parasitic cell cycle [88,139]. *Pf*nek-1 and the three Aurora-related kinases are likely essential for erythrocytic schizogony, while *Pf*nek-2 and *Pf*nek-4 are essential for sexual development of the parasite [139].

**Figure 22:**
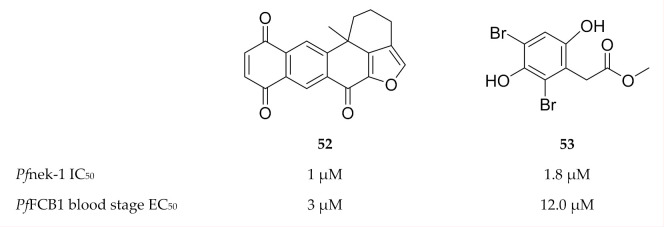
Structures of marine sponge compounds as inhibitors of *Pf*nek-1. *Pf*FCB1: *Plasmodium falciparum* FCB1 strain.

With regards to inhibitor development, compounds (e.g., **52** and **53**, Figure 22) isolated from marine sponges were found to inhibit *Pf*nek-1 and demonstrate in vitro *P. falciparum* activity [140,141,142]. Human Aurora kinase inhibitors also demonstrated antiplasmodial activity in vitro; however, the activity is yet to be attributed to plasmodial Aurora-related kinase inhibition [143].

## 7. Phosphoinositide Lipid Kinases (PIKs)

Five *P. falciparum* PIKs have been identified that are phylogenetically divergent from human PIKs [144]. The phosphoinositide 3-kinase (*Pf*PI3K) and phosphatidylinositol 4-kinase (*Pf*PI4K) homologues are both essential for parasite survival and most likely involved in cellular signalling and trafficking [145,146,147].

### 7.1. PIK Inhibitor Development

#### 7.1.1. PI3K

**Figure 23:**
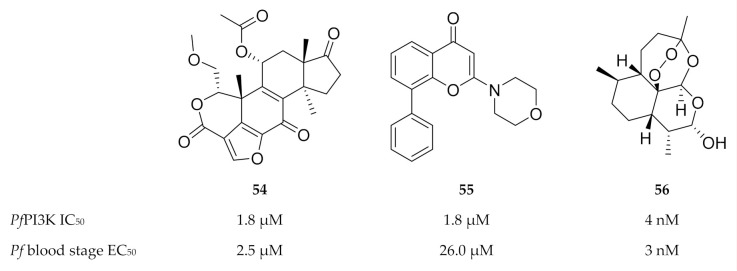
Structures of wortmannin, LY294002 and dihydroartemisinin.

To date, only two compounds have been identified for studying the functions of *Pf*PI3K. The mammalian PI3K inhibitors (Figure 23) wortmannin (**54**) and LY294002 (**55**) both inhibit *Pf*PI3K and blood-stage *P. falciparum* parasite growth [146,147]. *Pf*PI3K was also identified as a target of dihydroartemisinin (**56**, Figure 23) during the early ring stages [148].

#### 7.1.2. PI4K

Considerable progress has been made with regards to inhibitor development for PI4K, with one candidate progressing into clinical trial evaluation. The imidazopyridine/ pyrazine/ pyridazine class [145,149,150,151,152], the aminopyridine/pyrazine class [153,154,155,156] and the bipyridine sulfonamide [157] scaffolds have been evaluated for PI4K inhibition.

**Figure 24:**
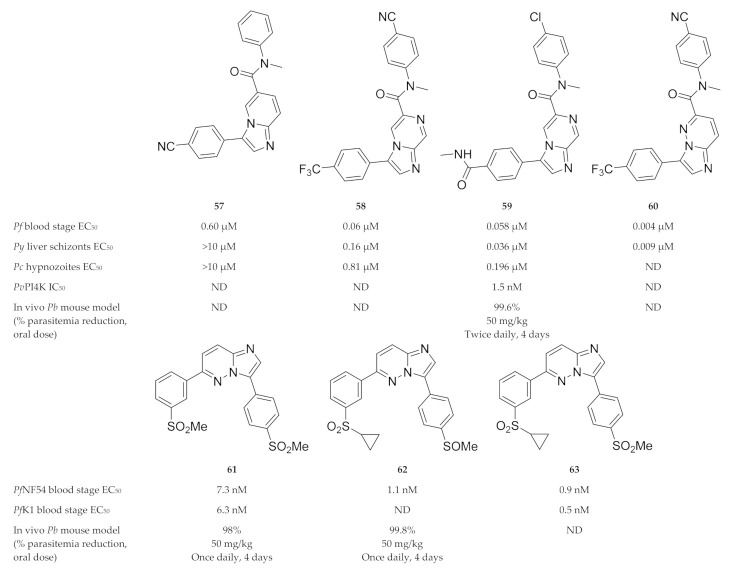
Imidazopyridine-, pyrazine- and pyridazine-based compounds as inhibitors of plasmodial PI4K.

A phenotypic screening identified compound **57** (Figure 24), which demonstrated activity against *P. falciparum* blood-stage parasites but was inactive against *P. yoelii* (*Py*) and *P. cynomolgi* (*Pc*) liver-stage parasites [152]. Liver-stage activity was acquired by replacing the imidazopyridine core of compound **57** with an imidazopyrazine core (KAI407, **58**, Figure 24). From a series of imidazopyrazines, compound **59** (KDU691, Figure 24) had optimal antimalarial activity (blood and liver stages) and physicochemical properties, which translated into in vivo efficacy against *P. berghei*-infected mice [152]. McNamara and co-workers [145] further demonstrated that compound **59** reduced liver- and blood-stage parasites, gametocyte viability and transmission to the mosquito vector for multiple *Plasmodium* species. Plasmodial PI4K was identified as the direct target of imidazopyrazine compounds [145].

Le Manach and co-workers [150] synthesised a series of imidazopyrazines based on structures of hits identified during a screening campaign. Compound **61** (Figure 24) was highly active against drug-sensitive (NF54) and -resistant (K1) *P. falciparum* strains and reduced parasitemia by 98% in *P. berghei*-infected mice (4 × 50 mg/kg). However, compound **61** failed to produce significant in vivo efficacy at lower doses, displayed poor solubility and showed activity towards hERG. Further optimisation of this scaffold led to compound **62** (Figure 24), which was completely curative in *P. berghei*-infected mice (4 × 50 mg/kg) and retained high in vivo efficacy at lower doses [151]. Compound **62** acted as a prodrug which was rapidly metabolised to the highly active sulfone (compound **63**, Figure 24) in vivo.

**Figure 25:**
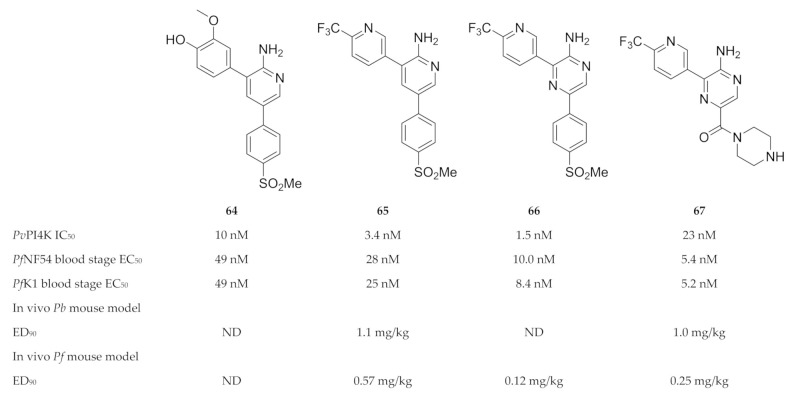
Structures and biological data of aminopyridine- and pyrazine-based compounds as inhibitors of plasmodial PI4K.

Compound **64** (Figure 25) was identified during a high-throughput screening campaign against drug-sensitive (NF54) and -resistant (K1) *P. falciparum* strains [155]. Optimisation of the 2-aminopyridine scaffold resulted in compound **65** (MMV390048 or MMV048, Figure 25), which demonstrated in vitro and in vivo activity against the liver, blood and sexual (gametocyte) stages of the parasite [155,158]. Whole-genome screening of MMV048-resistant *P. falciparum* strains and chemoproteomic profiling identified plasmodial PI4K as the target of MMV048 [158]. MMV048 showed high selectivity over human and other plasmodial kinases, a good ADME profile and an acceptable preclinical safety profile in various animal species (mice, rats, dogs and monkeys) [155,158]. Phase I clinical trials for MMV048 were recently completed (ClinicalTrials.gov: NCT02230579; NCT02281344; NCT02554799) [159]. A single oral dose of up to 120 mg was generally well tolerated in healthy volunteers and adverse events were mild to moderate. Treatment with 20 mg of MMV048 initially reduced parasitemia in volunteers with induced *P. falciparum* blood-stage malaria; however, recrudescence occurred 2 to 7 days after treatment. Formulation influences the pharmacodynamic profile of MMV048, with the tablet formulation resulting in significantly less variability than the powder-in-a-bottle formulation. MMV048 progressed to phase 2a clinical trials in 2017, where its activity was evaluated in Ethiopian adults with either uncomplicated *P. falciparum* or *P. vivax* infection (ClinicalTrials.gov: NCT02880241).

Younis and co-workers [156] replaced the 2-aminopyridine core of MMV048 with a 2-aminopyrazine ring (compound **66**, Figure 25), which improved the in vitro antimalarial activity but had poor solubility. In an attempt to improve the aqueous solubility, the methyl sulfonyl group of compound **66** was replaced with a piperazinyl carboxamide (UCT943, **67**, Figure 25) [154]. While retaining PI4K selectivity, UCT943 demonstrated improved solubility and potency against all life cycle stages compared to the clinical candidate, MMV048 [154,160]. The in vivo efficacy of UCT943 in a *P. falciparum*-infected NSG mouse model was also 2-fold more potent than that of MMV048 [160]. Gibhard and co-workers [153] also explored the option of using the more soluble sulfoxide analogue of compound **66** as a prodrug to improve drug exposure in vivo. In a *P. falciparum*-infected NSG mouse model, the sulfoxide was rapidly absorbed and converted to its sulfone analogue (compound **66**), which resulted in higher exposure compared to when the sulfone was administered.

**Figure 26:**
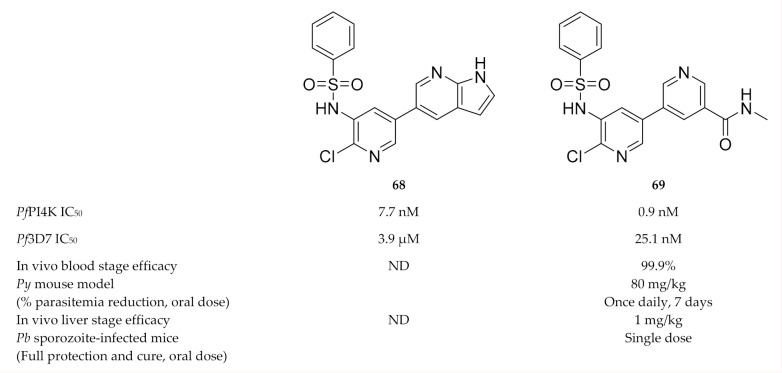
Bipyridine sulfonamide based compounds as inhibitors of *Pf*PI4K.

Hit compound **68** (Figure 26), discovered during a phenotypic screening campaign, displayed weak in vitro activity against *P. falciparum* blood-stage parasites (EC_50_: 3.9 µM) but had potent *Pf*PI4K activity (IC_50_: 7.7 nM) [157]. Systematic optimisation of the bipyridine sulfonamide scaffold led to compound **69** (Figure 26) which was selective for *Pf*PI4K and had potent activity against several drug-sensitive and -resistant *P. falciparum* strains. Compound **69** showed in vivo blood-stage efficacy (99.9% reduction in parasitemia at 80 mg/kg for 7 days) in a *P. yoelii*-infected mouse model and liver-stage efficacy (1 mg/kg, single dose) in a *P. berghei*-infected mouse model.

**Figure 27:**
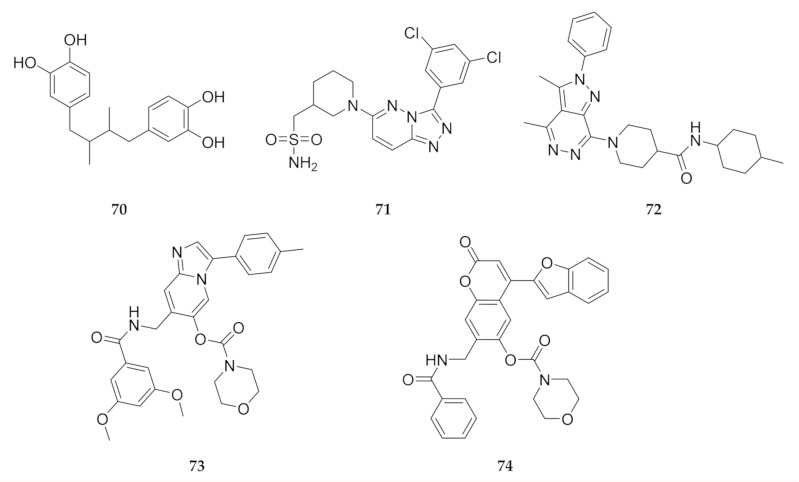
Hits identified during in silico screening against *Pf*PI4K homology models.

In silico screening campaigns against *Pf*PI4K homology models have identified a number of virtual hits that can be used as potential starting points for drug development [149,161,162]. Compounds **70**–**74** (Figure 27) all have good ADMET properties and form strong interactions with the ATP-binding site of *Pf*PI4K. Compounds **71** and **72** also demonstrated selectivity for *Pf*PI4K over the human PI4KB orthologue [149].

## 8. Orphan Kinases

Orphan kinases are particularly attractive drug targets as they do not have orthologues in the human host [11]. Some of the orphan kinases that have been characterised for *P. falciparum* include protein kinase 7 (*Pf*PK7), protein kinase 9 (*Pf*PK9) and the FIKK family [163].

*Pf*PK7 is a composite kinase that displays homology to the mitogen-activated protein kinase (MAPKK) family in its C-terminal region and fungal PKA homology in its N-terminal region [164]. Despite its homology, *Pf*PK7 is unlikely to be a functional MAPKK orthologue as the typical MAPKK activation site is absent and it is unable to phosphorylate the two MAPK orthologues (*Pf*map-1 and *Pf*map-2) in vitro [164]. *Pf*PK7 is expressed in asexual liver- and blood-stage parasites as well as in gametocytes and is localised in the cytoplasm [164]. Disruption of the *pfpk7* gene decreases the growth rate of erythrocytic parasites and drastically reduces the parasite’s ability to produce oocysts during the sexual stage [165].

*Pf*PK9 clusters at the base of the CDPK and AGC family branches but does not associate with either of the two groups [13]. *Pf*PK9 is essential for *P. falciparum* parasite viability [22,166] and is expressed during the late ring stages as well as the schizont stage where it exhibits maximal expression [167]. During the ring stages, *Pf*PK9 is localised to the parasitophorous vacuolar membrane, which acts as the interface between the parasite and the cytoplasm of the erythrocyte. As the parasite matures into schizonts, the localisation of *Pf*PK9 shifts to the parasite’s plasma membrane [167]. This suggests that *Pf*PK9 is involved in signal transduction between the cytosol of the parasite and the intraerythrocytic environment. Thus far, only one downstream target has been identified for *Pf*PK9, namely E2 ubiquitin-conjugating enzyme 13 (*Pf*UBC13) [167]. *Pf*UBC13 is an orthologue of eukaryotic UBC13 which is involved in the attachment of lysine 63 (K63)-linked polyubiquitin chains to target proteins. This modulates the activity of various cellular processes such as DNA repair and immune responses.

The FIKK family, a group of serine/threonine kinases specific to apicomplexan parasites, is named after the phenylalanine (F)–isoleucine (I)–lysine (K)–lysine (K) motif located in the *N*-terminal region of their kinase domains. While most *Plasmodium* species only have a single FIKK kinase, 20 FIKK kinase members have been identified for *P. falciparum* [168]. Although the biological functions of this group of kinases are still unclear, evidence suggests that most FIKK kinases are involved in erythrocyte remodelling during infection [169,170]. Studies have identified nine FIKK kinases that are exported via the Maurer’s clefts to the erythrocytic membrane, where remodelling occurs [169,171]. Disruption of individual genes encoding for *Pf*FIKK4.2, *Pf*FIKK7.1 or *Pf*FIKK12 significantly altered erythrocytic membrane rigidity and phosphorylation of certain cytoskeletal membrane proteins [170,172]. One such erythrocytic cytoskeletal protein, dematin, was also identified as a potential substrate for *Pf*FIKK4.1 [173].

### 8.1. Inhibitor Development for Orphan Kinases

#### 8.1.1. *Pf*PK7

**Figure 28:**
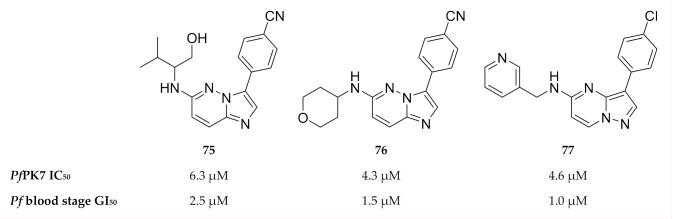
Imidazopyridine- and pyrazolopyrimidine-based compounds as inhibitors of *Pf*PK7.

As *Pf*PK7 plays a role in both the erythrocytic and sexual stages, *Pf*PK7 inhibitors could possibly decrease parasite virulence and act as transmission-blocking agents. This possibility makes *Pf*PK7 an interesting target for drug development. A number of established kinase inhibitors, including a MAPKK inhibitor (U0126) and PKA inhibitors (H89 & PKI), had no activity against recombinant *Pf*PK7 (IC_50_ >100 µM) [164]. A high-throughput screening campaign identified imidazopyridines (compounds **75** and **76**, Figure 28) and pyrazolopyrimidine (compound **77**, Figure 28) with *Pf*PK7 and in vitro activity in the low micromolar range [174]. However, these compounds also inhibited a number of other kinases in the low micromolar range.

**Figure 29:**
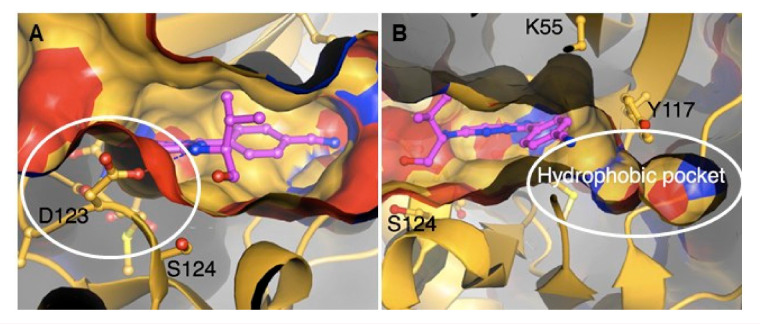
Structure of *Pf*PK7 in complex with compound **75**. (**A**) The protruding aspartic acid residue (D123) blocks access to the C-terminal domain surface of the ATP-binding site. (**B**) The hydrophobic pocket located at the back of the ATP-binding site. (Figure reproduced from [174] with permission from Elsevier.)

The crystal structures of *Pf*PK7 in complex with adenylylimidodiphosphate (an ATP analogue, PBD: 2PML), compound **75** (PBD: 2PMN) and hymenialdisine (PBD: 2PMO) were elucidated. These structures highlighted some atypical features that are specifically relevant to drug discovery: Firstly, an aspartic acid residue (D123) in the hinge region protrudes to block access to the C-terminal domain surface in the ATP-binding site (Figure 29A). This structural impediment explains the inactivity of most established protein kinase inhibitors towards *Pf*PK7. Secondly, a hydrophobic pocket was identified in the back of the ATP-binding site, which can be exploited for designing *Pf*PK7-selective drugs (Figure 29B).

**Figure 30:**
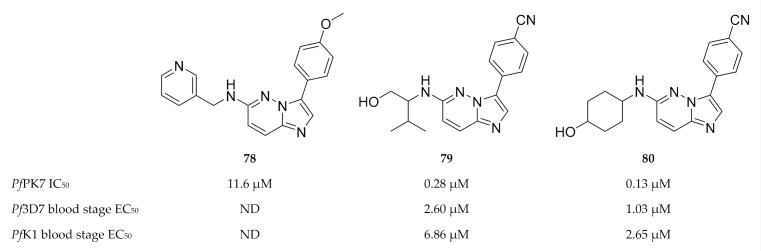
Imidazopyridazine-based compounds as inhibitors of *Pf*PK7.

Another high-throughput screening campaign identified a number of imidazopyridazines (e.g., **78**, Figure 30) as weak *Pf*PK7 inhibitors [175]. Using the crystal structure data from the previous study [174], Bouloc and co-workers [175] aimed to improve the potency of the imidazopyridazine scaffold by varying the aryl and amine substituents of **78**. This ultimately led to compounds **79** and **80** which demonstrated improved *Pf*PK7 activity (IC_50_: 0.28 µM and 0.13 µM, respectively). These compounds also showed antiplasmodial activity against drug-sensitive (3D7) and -resistant (K1) strains of *P. falciparum* without significant cytotoxicity. However, these compounds were also unselective and inhibited several other kinases.

**Figure 31:**
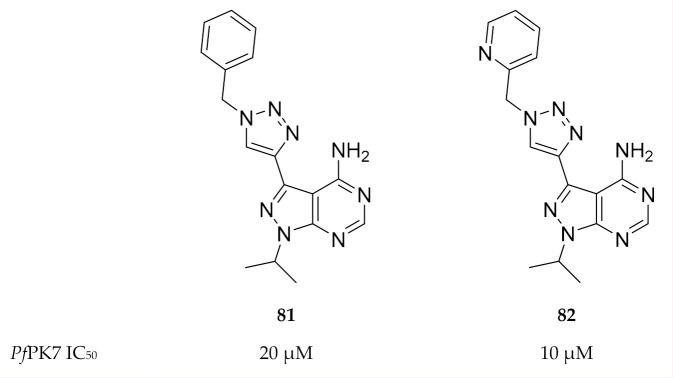
Structures of pyrazolopyrimidine-based compounds with a bent geometry.

Klein and co-workers [176] aimed to design *Pf*PK7 inhibitors that exploit the hydrophobic pocket in the ATP-binding site. They designed two series of pyrazolopyrimidines with 1- and 4-substituted triazole rings at the 3-position of the pyrazole ring. The substituted triazoles were designed with a “bent” geometry that would allow the inhibitor to interact with the hydrophobic pocket. Two compounds (**81** and **82**, Figure 31) demonstrated *Pf*PK7 activity in micromolar concentrations (IC_50_: 20 and 10 µM, respectively). Docking studies with compound **81** indicated binding interactions similar to those seen with the ATP analogue but with additional interaction of the 4-phenyl-(1,2,3-triazol-1-yl) moiety with the hydrophobic pocket.

#### 8.1.2. *Pf*PK9

**Figure 32:**
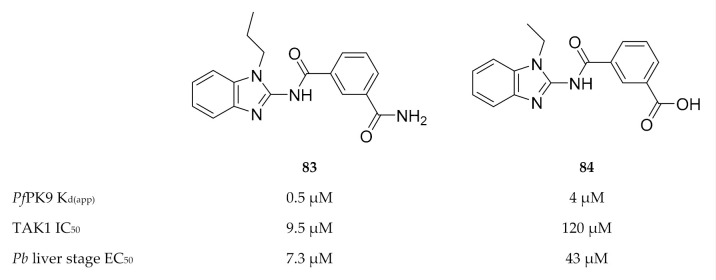
Structures and biological data of takinib and its analogue. K_D(app)_: apparent dissociation constant.

Only one study has been published thus far with regards to inhibitor development for *Pf*PK9. Screening of a kinase-targeted library against *Pf*PK9 identified takinib (**83**, Figure 32), which demonstrated low micromolar binding affinity (K_d(app)_: 0.46 µM) for *Pf*PK9 [177]. Takinib is a potent inhibitor (IC_50_: 9.5 nM, [178]) of human mitogen-activated protein kinase kinase kinase 7 (MAP3K7, or more commonly referred to as TAK1). TAK1′s activity is regulated through K63-ubiquitination by human ubiquitin-conjugating enzyme, UBC13 [179]. Treatment of *P. falciparum*-infected erythrocytes with takinib resulted in a dose-dependent reduction in K63-ubiquitin levels, confirming that *Pf*PK9 regulates the activity of *Pf*UBC13 in vivo [167]. In order to achieve *Pf*PK9 selectivity over TAK1, a series of takinib analogues was developed. From this series, compound **84** (Figure 32) was identified as a *Pf*PK9-selective inhibitor (K_d(app)_: 4.1 µM, 8.9-fold less potent than takinib) with antiparasitic activity against liver-stage *P. berghei* parasites (EC_50_ = 43 µM) and no significant hepatocyte cytotoxicity. Compound **84** also showed a similar decrease in K63-linked ubiquitin levels in *P. falciparum*-infected erythrocytes, as seen with takinib. Interestingly, takinib and compound **84** both induced an unusual phenotype in liver-stage parasites. Liver-stage drugs generally decrease the size and/or number of parasites. However, when liver-stage *P. berghei* parasites were treated with 10 µM of either takinib or compound **84**, the parasite size increased while the number of parasites remained unchanged. Treatment with 30 µM of either takinib or compound **84** also increased the parasite size but simultaneously decreased the number of parasites. This suggests a unique mechanism of action for these inhibitors, which has potential in new antimalarial drug development.

#### 8.1.3. FIKKs

To date, five *P. falciparum* FIKK kinase members have been identified as essential for parasite survival, three of which are exported to the erythrocytic membrane (*Pf*FIKK9.1, *Pf*FIKK10.1 and *Pf*FIKK10.2) and two of which are localised within the parasite (*Pf*FIKK3 and *Pf*FIKK9.5) [171]. The non-exported FIKK8 kinase was also demonstrated to be essential to *P. berghei* erythrocytic parasites [180]. FIKK8 is the only FIKK kinase member that is conserved in all *Plasmodium* species as well as other apicomplexan parasites [181].

**Figure 33:**
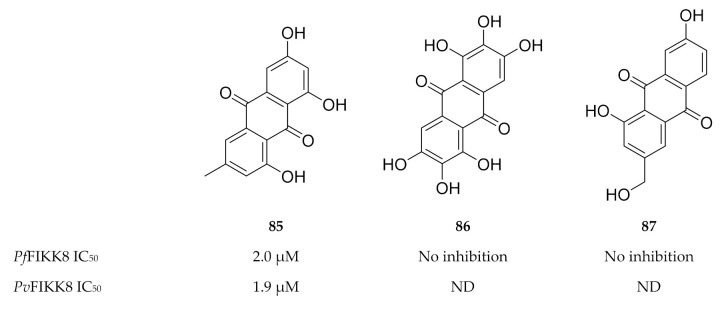
Structures of emodin, aloe emodin and rufigallol.

Interestingly, FIKK kinases tend to have small gatekeeper residues [180,182], a characteristic that is rarely seen in human serine/threonine kinases but is more common to human tyrosine kinases [183]. Not surprisingly, a screening campaign identified a number of tyrosine kinase inhibitors that block FIKK kinase activity [182,184]. One such tyrosine inhibitor, an anthraquinone named emodin (**85**, Figure 33), demonstrated in vitro activity against various *P. falciparum* strains (IC_50_ values of approximately 13 µM) [185] as well as activity against *Pf*FIKK8 (IC_50_: 2 µM) and the *P. vivax* orthologue (*Pv*FIKK8; IC_50_: 1.9 µM) [182,184]. Structurally related analogues of emodin (Figure 33), aloe emodin (**86**) and rufigallol (**87**), did not demonstrate any significant activity against *Pf*FIKK8 [184].

## 9. Summary

Recurring antimalarial drug resistance necessitates the development of new antimalarial drugs with different chemical scaffolds and modes of action. Plasmodial kinases were identified as promising targets for next-generation antimalarial drug development. To date, significant progress has been made towards characterisation and small-molecule inhibitor development for plasmodial kinases. Various plasmodial kinases have been validated as essential for one or multiple stages of the parasite’s life cycle. Therefore, targeting plasmodial kinases could result in new drugs for chemoprevention and transmission-blocking, which will contribute to malaria elimination.

Plasmodial kinase inhibitors have been successfully identified by means of phenotypic and target-based screening approaches. The expression of several plasmodial kinases as active recombinant enzymes has facilitated crystallography and target-based medicinal chemistry efforts. Knowledge gained from developing human kinase inhibitors has also significantly contributed to plasmodial kinase inhibitor development. A number of potential scaffolds were identified through either phenotypic or target-based screening of human kinase inhibitor libraries.

Inhibitor promiscuity is always a concern when dealing with a conserved group of targets such as the kinases. Therefore, it is important to cross-screen potential plasmodial kinase inhibitors against human kinase panels. Inhibitors that target multiple plasmodial kinases could be beneficial as this ability would limit the risk of drug resistance, provided that this promiscuity does not also affect host kinases. Overall, studies targeting plasmodial kinases have demonstrated that selectivity over host kinases is an achievable goal.

Although a significant amount of work still needs to be done in terms of fully understanding the functions, stage specificity and interactions of plasmodial kinases, this group shows promise for future antimalarial drug development.

## References

[1] WHO (2019). World Malaria Report. https://www.who.int/malaria/publications/world_malaria_report/en/.

[2] WHO The Potential Impact of Health Service Disruption on the Burden of Malaria: A Modelling Analysis for Countries in Sub-Saharan Africa. https://www.who.int/publications/i/item/the-potential-impact-of-health-service-disruptions-on-the-burden-of-malaria.

[3] Nghochuzie N.N., Olwal C.O., Udoakang A.J., Amenga-Etego L.N., Amambua-Ngwa A. (2020). Pausing the Fight Against Malaria to Combat the COVID-19 Pandemic in Africa: Is the Future of Malaria Bleak?. Front. Microbiol..

[4] WHO Status Report on Artemisinin Resistance and Artemisinin-Based Combination Therapy Efficacy. https://www.who.int/malaria/areas/drug_resistance/updates/en/.

[5] Noisang C., Prosser C., Meyer W., Chemoh W., Ellis J., Sawangjaroen N., Lee R. (2019). Molecular detection of drug resistant malaria in Southern Thailand. Malar J..

[6] Yadav D.K., Kumar S., Teli M.K., Yadav R., Chaudhary S. (2019). Molecular Targets for Malarial Chemotherapy: A Review. Curr. Top. Med. Chem..

[7] Rout S., Mahapatra R.K. (2019). Plasmodium falciparum: Multidrug resistance. Chem. Biol. Drug Des..

[8] Burrows J.N., Duparc S., Gutteridge W.E., Hooft van Huijsduijnen R., Kaszubska W., Macintyre F., Mazzuri S., Mohrle J.J., Wells T.N.C. (2017). New developments in anti-malarial target candidate and product profiles. Malar. J..

[9] Doerig C. (2004). Protein kinases as targets for anti-parasitic chemotherapy. Biochim. Biophys. Acta.

[10] Doerig C., Abdi A., Bland N., Eschenlauer S., Dorin-Semblat D., Fennell C., Halbert J., Holland Z., Nivez M.P., Semblat J.P. (2010). Malaria: Targeting parasite and host cell kinomes. Biochim. Biophys. Acta.

[11] Doerig C., Meijer L. (2007). Antimalarial drug discovery: Targeting protein kinases. Expert. Opin. Targets.

[12] Srinivasan A.N., Krupa A. (2005). A genomic perspective of protein kinases in *Plasmodium falciparum*. Proteins.

[13] Ward P., Equinet L., Packer J., Doerig C. (2004). Protein kinases of the human malaria parasite *Plasmodium falciparum*: The kinome of a divergent eukaryote. Bmc Genom..

[14] Cabrera D.G., Horatscheck A., Wilson C.R., Basarab G., Eyermann C.J., Chibale K. (2018). Plasmodial kinase inhibitors: License to cure?. J. Med. Chem..

[15] Billker O., Lourido S., Sibley L.D. (2009). Calcium-dependent signaling and kinases in apicomplexan parasites. Cell Host Microbe.

[16] Ghartey-Kwansah G., Yin Q., Li Z., Gumpper K., Sun Y., Yang R., Wang D., Jones O., Zhou X., Wang L. (2020). Calcium-dependent Protein Kinases in Malaria Parasite Development and Infection. Cell Transpl..

[17] Hui R., El Bakkouri M., Sibley L.D. (2015). Designing selective inhibitors for calcium-dependent protein kinases in apicomplexans. Trends Pharm. Sci..

[18] Green J.L., Rees-Channer R.R., Howell S.A., Martin S.R., Knuepfer E., Taylor H.M., Grainger M., Holder A.A. (2008). The motor complex of *Plasmodium falciparum*: Phosphorylation by a calcium-dependent protein kinase. J. Biol. Chem..

[19] Kato N., Sakata T., Breton G., Le Roch K.G., Nagle A., Andersen C., Bursulaya B., Henson K., Johnson J., Kumar K.A. (2008). Gene expression signatures and small-molecule compounds link a protein kinase to *Plasmodium falciparum* motility. Nat. Chem. Biol..

[20] Bansal A., Singh S., More K.R., Hans D., Nangalia K., Yogavel M., Sharma A., Chitnis C.E. (2013). Characterization of *Plasmodium falciparum* calcium-dependent protein kinase 1 (PfCDPK1) and its role in microneme secretion during erythrocyte invasion. J. Biol. Chem..

[21] Bansal A., Molina-Cruz A., Brzostowski J., Liu P., Luo Y., Gunalan K., Li Y., Ribeiro J.M.C., Miller L.H. (2018). PfCDPK1 is critical for malaria parasite gametogenesis and mosquito infection. Proc. Natl. Acad. Sci. USA.

[22] Solyakov L., Halbert J., Alam M.M., Semblat J.P., Dorin-Semblat D., Reininger L., Bottrill A.R., Mistry S., Abdi A., Fennell C. (2011). Global kinomic and phospho-proteomic analyses of the human malaria parasite *Plasmodium falciparum*. Nat. Commun..

[23] Green J.L., Moon R.W., Whalley D., Bowyer P.W., Wallace C., Rochani A., Nageshan R.K., Howell S.A., Grainger M., Jones H.M. (2015). Imidazopyridazine Inhibitors of *Plasmodium falciparum* Calcium-Dependent Protein Kinase 1 Also Target Cyclic GMP-Dependent Protein Kinase and Heat Shock Protein 90 to Kill the Parasite at Different Stages of Intracellular Development. Antimicrob. Agents Chemother..

[24] Kumar S., Kumar M., Ekka R., Dvorin J.D., Paul A.S., Madugundu A.K., Gilberger T., Gowda H., Duraisingh M.T., Keshava Prasad T.S. (2017). PfCDPK1 mediated signaling in erythrocytic stages of *Plasmodium falciparum*. Nat. Commun..

[25] Sebastian S., Brochet M., Collins M.O., Schwach F., Jones M.L., Goulding D., Rayner J.C., Choudhary J.S., Billker O. (2012). A *Plasmodium* calcium-dependent protein kinase controls zygote development and transmission by translationally activating repressed mRNAs. Cell Host Microbe.

[26] Bansal A., Molina-Cruz A., Brzostowski J., Mu J., Miller L.H. (2017). *Plasmodium falciparum* Calcium-Dependent Protein Kinase 2 Is Critical for Male Gametocyte Exflagellation but Not Essential for Asexual Proliferation. mBio.

[27] Kato K., Sudo A., Kobayashi K., Sugi T., Tohya Y., Akashi H. (2009). Characterization of *Plasmodium falciparum* calcium-dependent protein kinase 4. Parasitol. Int..

[28] Li J., Baker D.A., Cox L.S. (2000). Sexual stage-specific expression of a third calcium-dependent protein kinase from *Plasmodium falciparum*. Biochim. Biophys. Acta.

[29] Govindasamy K., Jebiwott S., Jaijyan D.K., Davidow A., Ojo K.K., Van Voorhis W.C., Brochet M., Billker O., Bhanot P. (2016). Invasion of hepatocytes by *Plasmodium* sporozoites requires cGMP-dependent protein kinase and calcium dependent protein kinase 4. Mol. Microbiol..

[30] Ishino T., Orito Y., Chinzei Y., Yuda M. (2006). A calcium-dependent protein kinase regulates *Plasmodium* ookinete access to the midgut epithelial cell. Mol. Microbiol..

[31] Siden-Kiamos I., Ecker A., Nyback S., Louis C., Sinden R.E., Billker O. (2006). *Plasmodium berghei* calcium-dependent protein kinase 3 is required for ookinete gliding motility and mosquito midgut invasion. Mol. Microbiol..

[32] Absalon S., Blomqvist K., Rudlaff R.M., DeLano T.J., Pollastri M.P., Dvorin J.D. (2018). Calcium-dependent protein kinase 5 is required for release of egress-specific organelles in *Plasmodium falciparum*. mBio.

[33] Dvorin J.D., Martyn D.C., Patel S.D., Grimley J.S., Collins C.R., Hopp C.S., Bright A.T., Westenberger S., Winzeler E., Blackman M.J. (2010). A plant-like kinase in *Plasmodium falciparum* regulates parasite egress from erythrocytes. Science.

[34] Kumar P., Tripathi A., Ranjan R., Halbert J., Gilberger T., Doerig C., Sharma P. (2014). Regulation of *Plasmodium falciparum* development by calcium-dependent protein kinase 7 (PfCDPK7). J. Biol. Chem..

[35] Coppi A., Tewari R., Bishop J.R., Bennett B.L., Lawrence R., Esko J.D., Billker O., Sinnis P. (2007). Heparan sulfate proteoglycans provide a signal to *Plasmodium* sporozoites to stop migrating and productively invade cells. Cell Host Microbe.

[36] Van Voorhis W.C., Doggett J.S., Parsons M., Hulverson M.A., Choi R., Arnold S.L.M., Riggs M.W., Hemphill A., Howe D.K., Mealey R.H. (2017). Extended-spectrum antiprotozoal bumped kinase inhibitors: A review. Exp. Parasitol..

[37] Bansal A., Ojo K.K., Mu J., Maly D.J., Van Voorhis W.C., Miller L.H. (2016). Reduced Activity of Mutant Calcium-Dependent Protein Kinase 1 Is Compensated in *Plasmodium falciparum* through the Action of Protein Kinase G. mBio.

[38] Lemercier G., Fernandez-Montalvan A., Shaw J.P., Kugelstadt D., Bomke J., Domostoj M., Schwarz M.K., Scheer A., Kappes B., Leroy D. (2009). Identification and characterization of novel small molecules as potent inhibitors of the plasmodial calcium-dependent protein kinase 1. Biochemistry.

[39] Ansell K.H., Jones H.M., Whalley D., Hearn A., Taylor D.L., Patin E.C., Chapman T.M., Osborne S.A., Wallace C., Birchall K. (2014). Biochemical and antiparasitic properties of inhibitors of the *Plasmodium falciparum* calcium-dependent protein kinase PfCDPK1. Antimicrob. Agents Chemother..

[40] Chapman T.M., Osborne S.A., Bouloc N., Large J.M., Wallace C., Birchall K., Ansell K.H., Jones H.M., Taylor D., Clough B. (2013). Substituted imidazopyridazines are potent and selective inhibitors of *Plasmodium falciparum* calcium-dependent protein kinase 1 (PfCDPK1). Bioorg. Med. Chem. Lett..

[41] Chapman T.M., Osborne S.A., Wallace C., Birchall K., Bouloc N., Jones H.M., Ansell K.H., Taylor D.L., Clough B., Green J.L. (2014). Optimization of an imidazopyridazine series of inhibitors of *Plasmodium falciparum* calcium-dependent protein kinase 1 (PfCDPK1). J. Med. Chem..

[42] Large J.M., Osborne S.A., Smiljanic-Hurley E., Ansell K.H., Jones H.M., Taylor D.L., Clough B., Green J.L., Holder A.A. (2013). Imidazopyridazines as potent inhibitors of *Plasmodium falciparum* calcium-dependent protein kinase 1 (PfCDPK1): Preparation and evaluation of pyrazole linked analogues. Bioorg. Med. Chem. Lett..

[43] Flaherty B.R., Ho T.G., Schmidt S.H., Herberg F.W., Peterson D.S., Kennedy E.J. (2019). Targeted Inhibition of *Plasmodium falciparum* Calcium-Dependent Protein Kinase 1 with a Constrained J Domain-Derived Disruptor Peptide. ACS Infect. Dis..

[44] Lima M.N.N., Cassiano G.C., Tomaz K.C.P., Silva A.C., Sousa B.K.P., Ferreira L.T., Tavella T.A., Calit J., Bargieri D.Y., Neves B.J. (2019). Integrative Multi-Kinase Approach for the Identification of Potent Antiplasmodial Hits. Front. Chem..

[45] Jain R., Gupta S., Munde M., Pati S., Singh S. (2020). Development of novel anti-malarial from structurally diverse library of molecules, targeting plant-like CDPK1, a multistage growth regulator of P. falciparum. Biochem. J..

[46] Cavagnino A., Rossi F., Rizzi M. (2011). The potent antiplasmodial calmodulin-antagonist trifluoperazine inhibits *Plasmodium falciparum* calcium-dependent protein kinase 4. Protein Pept. Lett..

[47] Huang W., Hulverson M.A., Zhang Z., Choi R., Hart K.J., Kennedy M., Vidadala R.S.R., Maly D.J., Van Voorhis W.C., Lindner S.E. (2016). 5-Aminopyrazole-4-carboxamide analogues are selective inhibitors of *Plasmodium falciparum* microgametocyte exflagellation and potential malaria transmission blocking agents. Bioorg. Med. Chem. Lett..

[48] Ojo K.K., Eastman R.T., Vidadala R., Zhang Z., Rivas K.L., Choi R., Lutz J.D., Reid M.C., Fox A.M., Hulverson M.A. (2014). A specific inhibitor of PfCDPK4 blocks malaria transmission: Chemical-genetic validation. J. Infect. Dis..

[49] Ojo K.K., Pfander C., Mueller N.R., Burstroem C., Larson E.T., Bryan C.M., Fox A.M., Reid M.C., Johnson S.M., Murphy R.C. (2012). Transmission of malaria to mosquitoes blocked by bumped kinase inhibitors. J. Clin. Investig..

[50] Vidadala R.S., Ojo K.K., Johnson S.M., Zhang Z., Leonard S.E., Mitra A., Choi R., Reid M.C., Keyloun K.R., Fox A.M. (2014). Development of potent and selective *Plasmodium falciparum* calcium-dependent protein kinase 4 (PfCDPK4) inhibitors that block the transmission of malaria to mosquitoes. Eur. J. Med. Chem..

[51] Perrin M.J., Subbiah R.N., Vandenberg J.I., Hill A.P. (2008). Human ether-a-go-go related gene (hERG) K+ channels: Function and dysfunction. Prog. Biophys. Mol. Biol..

[52] Aher R.B., Roy K. (2016). Exploring structural requirements for the inhibition of *Plasmodium falciparum* calcium-dependent protein kinase-4 (PfCDPK-4) using multiple in silico approaches. RSC Adv..

[53] Zhang Z., Ojo K.K., Vidadala R., Huang W., Geiger J.A., Scheele S., Choi R., Reid M.C., Keyloun K.R., Rivas K. (2014). Potent and selective inhibitors of CDPK1 from T. gondii and C. parvum based on a 5-aminopyrazole-4-carboxamide scaffold. ACS Med. Chem. Lett..

[54] Rout S., Mahapatra R.K. (2019). In silico analysis of plasmodium falciparum CDPK5 protein through molecular modeling, docking and dynamics. J. Biol..

[55] Dawn A., Singh S., More K.R., Siddiqui F.A., Pachikara N., Ramdani G., Langsley G., Chitnis C.E. (2014). The central role of cAMP in regulating *Plasmodium falciparum* merozoite invasion of human erythrocytes. PLoS Pathog.

[56] Brochet M., Collins M.O., Smith T.K., Thompson E., Sebastian S., Volkmann K., Schwach F., Chappell L., Gomes A.R., Berriman M. (2014). Phosphoinositide metabolism links cGMP-dependent protein kinase G to essential Ca(2)(+) signals at key decision points in the life cycle of malaria parasites. PLoS Biol..

[57] Vaid A., Sharma P. (2006). PfPKB, a protein kinase B-like enzyme from *Plasmodium falciparum*: II. Identification of calcium/calmodulin as its upstream activator and dissection of a novel signaling pathway. J. Biol. Chem..

[58] Wilde M., Triglia T., Marapana D., Thompson J.K., Kouzmitchev A.A., Bullen H.E., Gilson P.R., Cowman A.F., Tonkin C.J. (2019). Protein kinase A is essential for invasion of *Plasmodium falciparum* into human erythrocytes. MBio.

[59] Leykauf K., Treeck M., Gilson P.R., Nebl T., Braulke T., Cowman A.F., Gilberger T.W., Crabb B.S. (2010). Protein kinase a dependent phosphorylation of apical membrane antigen 1 plays an important role in erythrocyte invasion by the malaria parasite. PLoS Pathog.

[60] Patel A., Perrin A.J., Flynn H.R., Bisson C., Withers-Martinez C., Treeck M., Flueck C., Nicastro G., Martin S.R., Ramos A. (2019). Cyclic AMP signalling controls key components of malaria parasite host cell invasion machinery. PLoS Biol..

[61] Lasonder E., Green J.L., Camarda G., Talabani H., Holder A.A., Langsley G., Alano P. (2012). The *Plasmodium falciparum* schizont phosphoproteome reveals extensive phosphatidylinositol and cAMP-protein kinase A signaling. J. Proteome Res..

[62] Merckx A., Nivez M.P., Bouyer G., Alano P., Langsley G., Deitsch K., Thomas S., Doerig C., Egee S. (2008). *Plasmodium falciparum* regulatory subunit of cAMP-dependent PKA and anion channel conductance. PLoS Pathog.

[63] Beraldo F.H., Almeida F.M., da Silva A.M., Garcia C.R. (2005). Cyclic AMP and calcium interplay as second messengers in melatonin-dependent regulation of *Plasmodium falciparum* cell cycle. J. Cell Biol..

[64] Falae A., Combe A., Amaladoss A., Carvalho T., Menard R., Bhanot P. (2010). Role of *Plasmodium berghei* cGMP-dependent protein kinase in late liver stage development. J. Biol Chem..

[65] Alam M.M., Solyakov L., Bottrill A.R., Flueck C., Siddiqui F.A., Singh S., Mistry S., Viskaduraki M., Lee K., Hopp C.S. (2015). Phosphoproteomics reveals malaria parasite Protein Kinase G as a signalling hub regulating egress and invasion. Nat. Commun..

[66] Collins C.R., Hackett F., Strath M., Penzo M., Withers-Martinez C., Baker D.A., Blackman M.J. (2013). Malaria parasite cGMP-dependent protein kinase regulates blood stage merozoite secretory organelle discharge and egress. PLoS Pathog.

[67] Taylor H.M., McRobert L., Grainger M., Sicard A., Dluzewski A.R., Hopp C.S., Holder A.A., Baker D.A. (2010). The malaria parasite cyclic GMP-dependent protein kinase plays a central role in blood-stage schizogony. Eukaryot Cell.

[68] Vaid A., Thomas D.C., Sharma P. (2008). Role of Ca2+/calmodulin-PfPKB signaling pathway in erythrocyte invasion by *Plasmodium falciparum*. J. Biol. Chem..

[69] McRobert L., Taylor C.J., Deng W., Fivelman Q.L., Cummings R.M., Polley S.D., Billker O., Baker D.A. (2008). Gametogenesis in malaria parasites is mediated by the cGMP-dependent protein kinase. PLoS Biol..

[70] Moon R.W., Taylor C.J., Bex C., Schepers R., Goulding D., Janse C.J., Waters A.P., Baker D.A., Billker O. (2009). A cyclic GMP signalling module that regulates gliding motility in a malaria parasite. PLoS Pathog..

[71] Baker D.A., Stewart L.B., Large J.M., Bowyer P.W., Ansell K.H., Jimenez-Diaz M.B., El Bakkouri M., Birchall K., Dechering K.J., Bouloc N.S. (2017). A potent series targeting the malarial cGMP-dependent protein kinase clears infection and blocks transmission. Nat. Commun..

[72] Vanaerschot M., Murithi J.M., Pasaje C.F.A., Ghidelli-Disse S., Dwomoh L., Bird M., Spottiswoode N., Mittal N., Arendse L.B., Owen E.S. (2020). Inhibition of Resistance-Refractory P. falciparum Kinase PKG Delivers Prophylactic, Blood Stage, and Transmission-Blocking Antiplasmodial Activity. Cell Chem. Biol..

[73] Penzo M., de Las Heras-Duena L., Mata-Cantero L., Diaz-Hernandez B., Vazquez-Muniz M.J., Ghidelli-Disse S., Drewes G., Fernandez-Alvaro E., Baker D.A. (2019). High-throughput screening of the *Plasmodium falciparum* cGMP-dependent protein kinase identified a thiazole scaffold which kills erythrocytic and sexual stage parasites. Sci. Rep..

[74] Bakkouri M., Kouidmi I., Wernimont A.K., Amani M., Hutchinson A., Loppnau P., Kim J.J., Flueck C., Walker J.R., Seitova A. (2019). Structures of the cGMP-dependent protein kinase in malaria parasites reveal a unique structural relay mechanism for activation. Proc. Natl. Acad. Sci. USA.

[75] Large J.M., Birchall K., Bouloc N.S., Merritt A.T., Smiljanic-Hurley E., Tsagris D.J., Wheldon M.C., Ansell K.H., Coombs P.J., Kettleborough C.A. (2019). Potent inhibitors of malarial P. Falciparum protein kinase G: Improving the cell activity of a series of imidazopyridines. Bioorg. Med. Chem. Lett..

[76] Large J.M., Birchall K., Bouloc N.S., Merritt A.T., Smiljanic-Hurley E., Tsagris D.J., Wheldon M.C., Ansell K.H., Coombs P.J., Kettleborough C.A. (2019). Potent bicyclic inhibitors of malarial cGMP-dependent protein kinase: Approaches to combining improvements in cell potency, selectivity and structural novelty. Bioorg. Med. Chem. Lett..

[77] Matralis A.N., Malik A., Penzo M., Moreno I., Almela M.J., Camino I., Crespo B., Saadeddin A., Ghidelli-Disse S., Rueda L. (2019). Development of Chemical Entities Endowed with Potent Fast-Killing Properties against *Plasmodium falciparum* Malaria Parasites. J. Med. Chem..

[78] Tsagris D.J., Birchall K., Bouloc N., Large J.M., Merritt A., Smiljanic-Hurley E., Wheldon M., Ansell K.H., Kettleborough C., Whalley D. (2018). Trisubstituted thiazoles as potent and selective inhibitors of *Plasmodium falciparum* protein kinase G (PfPKG). Bioorg. Med. Chem. Lett..

[79] Biftu T., Feng D., Fisher M., Liang G.B., Qian X., Scribner A., Dennis R., Lee S., Liberator P.A., Brown C. (2006). Synthesis and SAR studies of very potent imidazopyridine antiprotozoal agents. Bioorg. Med. Chem. Lett..

[80] Biftu T., Feng D., Ponpipom M., Girotra N., Liang G.B., Qian X., Bugianesi R., Simeone J., Chang L., Gurnett A. (2005). Synthesis and SAR of 2,3-diarylpyrrole inhibitors of parasite cGMP-dependent protein kinase as novel anticoccidial agents. Bioorg. Med. Chem. Lett..

[81] Diaz C.A., Allocco J., Powles M.A., Yeung L., Donald R.G., Anderson J.W., Liberator P.A. (2006). Characterization of *Plasmodium falciparum* cGMP-dependent protein kinase (PfPKG): Antiparasitic activity of a PKG inhibitor. Mol. Biochem. Parasitol..

[82] Mahmood S.U., Cheng H., Tummalapalli S.R., Chakrasali R., Yadav Bheemanaboina R.R., Kreiss T., Chojnowski A., Eck T., Siekierka J.J., Rotella D.P. (2020). Discovery of isoxazolyl-based inhibitors of *Plasmodium falciparum* cGMP-dependent protein kinase. RSC Med. Chem..

[83] Buskes M.J., Harvey K.L., Prinz B., Crabb B.S., Gilson P.R., Wilson D.J., Abbott B.M. (2016). Exploration of 3-methylisoquinoline-4-carbonitriles as protein kinase A inhibitors of *Plasmodium falciparum*. Bioorg Med. Chem..

[84] Buskes M.J., Harvey K.L., Richards B.J., Kalhor R., Christoff R.M., Gardhi C.K., Littler D.R., Cope E.D., Prinz B., Weiss G.E. (2016). Antimalarial activity of novel 4-cyano-3-methylisoquinoline inhibitors against *Plasmodium falciparum*: Design, synthesis and biological evaluation. Org. Biomol. Chem..

[85] Kumar A., Vaid A., Syin C., Sharma P. (2004). PfPKB, a novel protein kinase B-like enzyme from *Plasmodium falciparum*: I. Identification, characterization, and possible role in parasite development. J. Biol. Chem..

[86] Littler D.R., Bullen H.E., Harvey K.L., Beddoe T., Crabb B.S., Rossjohn J., Gilson P.R. (2016). Disrupting the Allosteric Interaction between the *Plasmodium falciparum* cAMP-dependent Kinase and Its Regulatory Subunit. J. Biol. Chem..

[87] Sudo A., Kato K., Kobayashi K., Tohya Y., Akashi H. (2008). Susceptibility of *Plasmodium falciparum* cyclic AMP-dependent protein kinase and its mammalian homologue to the inhibitors. Mol. Biochem. Parasitol..

[88] Matthews H., Duffy C.W., Merrick C.J. (2018). Checks and balances? DNA replication and the cell cycle in *Plasmodium*. Parasit Vectors.

[89] Doerig C., Meijer L., Mottram J.C. (2002). Protein kinases as drug targets in parasitic protozoa. Trends Parasitol..

[90] Ross-Macdonald P.B., Graeser R., Kappes B., Franklin R., Williamson D.H. (1994). Isolation and expression of a gene specifying a cdc2-like protein kinase from the human malaria parasite *Plasmodium falciparum*. Eur. J. Biochem..

[91] Holton S., Merckx A., Burgess D., Doerig C., Noble M., Endicott J. (2003). Structures of P. falciparum PfPK5 test the CDK regulation paradigm and suggest mechanisms of small molecule inhibition. Structure.

[92] Deshmukh A.S., Agarwal M., Mehra P., Gupta A., Gupta N., Doerig C.D., Dhar S.K. (2015). Regulation of *Plasmodium falciparum* Origin Recognition Complex subunit 1 (PfORC1) function through phosphorylation mediated by CDK-like kinase PK5. Mol. Microbiol..

[93] Graeser R., Wernli B., Franklin R.M., Kappes B. (1996). *Plasmodium falciparum* protein kinase 5 and the malarial nuclear division cycles. Mol. Biochem. Parasitol..

[94] Li J., Robson K.J.H., Chen J., Targett G.A.T., Baker D. (1996). Pfmrk, a MO15-related protein kinase from *Plasmodium falciparum*. Eur. J. Biochem..

[95] Jirage D., Chen Y., Caridha D., O’Neil M.T., Eyase F., Witola W.H., Mamoun C.B., Waters N.C. (2010). The malarial CDK Pfmrk and its effector PfMAT1 phosphorylate DNA replication proteins and co-localize in the nucleus. Mol. Biochem. Parasitol..

[96] Halbert J., Ayong L., Equinet L., Le Roch K., Hardy M., Goldring D., Reininger L., Waters N., Chakrabarti D., Doerig C. (2010). A *Plasmodium falciparum* transcriptional cyclin-dependent kinase-related kinase with a crucial role in parasite proliferation associates with histone deacetylase activity. Eukaryot Cell.

[97] Doerig C., Doerig C., Horrocks P., Coyle J., Carlton J., Sultan A., Arnot D., Carter R. (1995). Pfcrk-1, a developmentally regulated cdc2-related protein kinase of *Plasmodium falciparum*. Mol. Biochem. Parasitol..

[98] Rangarajan R., Bei A., Henry N., Madamet M., Parzy D., Nivez M.P., Doerig C., Sultan A. (2006). Pbcrk-1, the *Plasmodium berghei* orthologue of P. falciparum cdc-2 related kinase-1 (Pfcrk-1), is essential for completion of the intraerythrocytic asexual cycle. Exp. Parasitol..

[99] Ganter M., Goldberg J.M., Dvorin J.D., Paulo J.A., King J.G., Tripathi A.K., Paul A.S., Yang J., Coppens I., Jiang R.H. (2017). *Plasmodium falciparum* CRK4 directs continuous rounds of DNA replication during schizogony. Nat. Microbiol..

[100] Bracchi-Ricard V., Barik S., Delvecchio C., Doerig C., Chakrabarti R., Chakrabarti D. (2000). PfPK6, a novel cyclin-dependent kinase/mitogen-activated protein kinase-related protein kinase from *Plasmodium falciparum*. Biochem. J..

[101] Dorin-Semblat D., Carvalho T.G., Nivez M.-P., Halbert J., Poullet P., Semblat J.-P., Goldring D., Chakrabarti D., Mehra P., Dhar S. (2013). An atypical cyclin-dependent kinase controls *Plasmodium falciparum* proliferation rate. Kinome.

[102] Chen Y., Jirage D., Caridha D., Kathcart A.K., Cortes E.A., Dennull R.A., Geyer J.A., Prigge S.T., Waters N.C. (2006). Identification of an effector protein and gain-of-function mutants that activate Pfmrk, a malarial cyclin-dependent protein kinase. Mol. Biochem. Parasitol..

[103] Le Roch K., Sestier C., Dorin D., Waters N., Kappes B., Chakrabarti D., Meijer L., Doerig C. (2000). Activation of a *Plasmodium falciparum* cdc2-related kinase by heterologous p25 and cyclin H. J. Biol. Chem..

[104] Eubanks A.L., Perkins M.M., Sylvester K., Ganley J.G., Posfai D., Sanschargrin P.C., Hong J., Sliz P., Derbyshire E.R. (2018). In silico Screening and Evaluation of *Plasmodium falciparum* Protein Kinase 5 (PK5) Inhibitors. ChemMedChem.

[105] Xiao Z., Waters N.C., Woodard C.L., Li Z., Li P. (2001). Design and synthesis of Pfmrk inhibitors as potential antimalarial agents. Bioorg. Med. Chem. Lett..

[106] Woodard C.L., Li Z., Kathcart A.K., Terrell J., Gerena L., Lopez-Sanchez M., Kyle D.E., Bhattacharjee A.K., Nichols D.A., Ellis W. (2003). Oxindole-based compounds are selective inhibitors of *Plasmodium falciparum* cyclin dependent protein kinases. J. Med. Chem..

[107] Geyer J.A., Keenan S.M., Woodard C.L., Thompson P.A., Gerena L., Nichols D.A., Gutteridge C.E., Waters N.C. (2009). Selective inhibition of Pfmrk, a *Plasmodium falciparum* CDK, by antimalarial 1,3-diaryl-2-propenones. Bioorg. Med. Chem. Lett..

[108] Akala H.M., Waters C.N., Yenesew A., Wanjala C., Akenga T.A. (2010). In vitro antiplasmodial and cyclin-dependent protein kinase (pfmrk) inhibitory activities of selected flavonoids in combination with chloroquine (CQ) and artemisinin. Res. Pharm. Biotechnol..

[109] Bhattacharjee A.K., Geyer J.A., Woodard C.L., Kathcart A.K., Nichols D.A., Prigge S.T., Li Z., Mott B.T., Waters N.C. (2004). A three-dimensional in silico pharmacophore model for inhibition of *Plasmodium falciparum* cyclin-dependent kinases and discovery of novel Pfmrk specific inhibitors. J. Med. Chem..

[110] Caridha D., Kathcart A.K., Jirage D., Waters N.C. (2010). Activity of substituted thiophene sulfonamides against malarial and mammalian cyclin dependent protein kinases. Bioorg. Med. Chem. Lett..

[111] Woodard C.L., Keenan S.M., Gerena L., Welsh W.J., Geyer J.A., Waters N.C. (2007). Evaluation of broad spectrum protein kinase inhibitors to probe the architecture of the malarial cyclin dependent protein kinase Pfmrk. Bioorg. Med. Chem. Lett..

[112] Bhattacharjee A.K., Hartell M.G., Nichols D.A., Hicks R.P., Stanton B., van Hamont J.E., Milhous W.K. (2004). Structure-activity relationship study of antimalarial indolo [2,1-b]quinazoline-6,12-diones (tryptanthrins). Three dimensional pharmacophore modeling and identification of new antimalarial candidates. Eur. J. Med. Chem..

[113] Dominguez J.N., Leon C., Rodrigues J., de Dominguez N.G., Gut J., Rosenthal P.J. (2005). Synthesis and evaluation of new antimalarial phenylurenyl chalcone derivatives. J. Med. Chem..

[114] Go M.L., Liu M., Wilairat P., Rosenthal P.J., Saliba K.J., Kirk K. (2004). Antiplasmodial chalcones inhibit sorbitol-induced hemolysis of *Plasmodium falciparum*-infected erythrocytes. Antimicrob. Agents Chemother..

[115] Ziegler H.L., Hansen H.S., Staerk D., Christensen S.B., Hagerstrand H., Jaroszewski J.W. (2004). The antiparasitic compound licochalcone a is a potent echinocytogenic agent that modifies the erythrocyte membrane in the concentration range where antiplasmodial activity is observed. Antimicrob. Agents Chemother..

[116] Joshi Y.N., Vinod P.S., Yemul V. (2017). Structural annotation and homology modeling of protein kinase 6 (PfPK6) from *Plasmodium falciparum*. IJSRST.

[117] Manhani K.K., Arcuri H.A., da Silveira N.J., Uchoa H.B., de Azevedo W.F., Canduri F. (2005). Molecular models of protein kinase 6 from *Plasmodium falciparum*. J. Mol. Model..

[118] Iwanaga T., Sugi T., Kobayashi K., Takemae H., Gong H., Ishiwa A., Murakoshi F., Recuenco F.C., Horimoto T., Akashi H. (2013). Characterization of *Plasmodium falciparum* cdc2-related kinase and the effects of a CDK inhibitor on the parasites in erythrocytic schizogony. Parasitol. Int..

[119] Dorin-Semblat D., Quashie N., Halbert J., Sicard A., Doerig C., Peat E., Ranford-Cartwright L., Doerig C. (2007). Functional characterization of both MAP kinases of the human malaria parasite *Plasmodium falciparum* by reverse genetics. Mol. Microbiol..

[120] Rangarajan R., Bei A.K., Jethwaney D., Maldonado P., Dorin D., Sultan A.A., Doerig C. (2005). A mitogen-activated protein kinase regulates male gametogenesis and transmission of the malaria parasite *Plasmodium berghei*. EMBO Rep..

[121] Brumlik M.J., Nkhoma S., Kious M.J., Thompson G.R., Patterson T.F., Siekierka J.J., Anderson T.J., Curiel T.J. (2011). Human p38 mitogen-activated protein kinase inhibitor drugs inhibit *Plasmodium falciparum* replication. Exp. Parasitol..

[122] Droucheau E., Primot A., Thomas V., Mattei D., Knockaert M., Richardson C., Sallicandro P., Alano P., Jafarshad A., Baratte B. (2004). *Plasmodium falciparum* glycogen synthase kinase-3: Molecular model, expression, intracellular localisation and selective inhibitors. Biochim. Biophys. Acta.

[123] Fugel W., Oberholzer A.E., Gschloessl B., Dzikowski R., Pressburger N., Preu L., Pearl L.H., Baratte B., Ratin M., Okun I. (2013). 3,6-Diamino-4-(2-halophenyl)-2-benzoylthieno[2,3-b]pyridine-5-carbonitriles are selective inhibitors of *Plasmodium falciparum* glycogen synthase kinase-3. J. Med. Chem..

[124] Kruggel S., Lemcke T. (2009). Comparative investigation of the ATP-binding site of human and plasmodial glycogen synthase kinase-3. QSAR Comb. Sci..

[125] Osolodkin D.I., Zakharevich N.V., Palyulin V.A., Danilenko V.N., Zefirov N.S. (2011). Bioinformatic analysis of glycogen synthase kinase 3: Human versus parasite kinases. Parasitology.

[126] Masch A., Nasereddin A., Alder A., Bird M.J., Schweda S.I., Preu L., Doerig C., Dzikowski R., Gilberger T.W., Kunick C. (2019). Structure-activity relationships in a series of antiplasmodial thieno[2,3-b]pyridines. Malar. J..

[127] Moolman C., Van der Sluis R., Beteck R.M., Legoabe L.J. (2020). Exploration of benzofuran-based compounds as potent and selective *Plasmodium falciparum* glycogen synthase kinase-3 (PfGSK-3) inhibitors.

[128] Agarwal S., Kern S., Halbert J., Przyborski J.M., Baumeister S., Dandekar T., Doerig C., Pradel G. (2011). Two nucleus-localized CDK-like kinases with crucial roles for malaria parasite erythrocytic replication are involved in phosphorylation of splicing factor. J. Cell Biochem..

[129] Kern S., Agarwal S., Huber K., Gehring A.P., Strodke B., Wirth C.C., Brugl T., Abodo L.O., Dandekar T., Doerig C. (2014). Inhibition of the SR protein-phosphorylating CLK kinases of *Plasmodium falciparum* impairs blood stage replication and malaria transmission. PLoS ONE.

[130] Talevich E., Mirza A., Kannan N. (2011). Structural and evolutionary divergence of eukaryotic protein kinases in Apicomplexa. BMC Evol. Biol..

[131] Schneider M., Hsiao H.H., Will C.L., Giet R., Urlaub H., Luhrmann R. (2010). Human PRP4 kinase is required for stable tri-snRNP association during spliceosomal B complex formation. Nat. Struct. Mol. Biol..

[132] Dixit A., Singh P.K., Sharma G.P., Malhotra P., Sharma P. (2010). PfSRPK1, a novel splicing-related kinase from *Plasmodium falciparum*. J. Biol. Chem..

[133] Ngwa C.J., Scheuermayer M., Mair G.R., Kern S., Brügl T., Wirth C.C., Aminake M.N., Wiesner J., Fischer R., Vilcinskas A. (2013). Changes in the transcriptome of the malaria parasite *Plasmodium falciparum* during the initial phase of transmission from the human to the mosquito. BMC Genom..

[134] Eshar S., Allemand E., Sebag A., Glaser F., Muchardt C., Mandel-Gutfreund Y., Karni R., Dzikowski R. (2012). A novel *Plasmodium falciparum* SR protein is an alternative splicing factor required for the parasites’ proliferation in human erythrocytes. Nucleic. Acids Res..

[135] Alam M.M., Sanchez-Azqueta A., Janha O., Flannery E.L., Mahindra A., Mapesa K., Char A.B., Sriranganadane D., Brancucci N.M.B., Antonova-Koch Y. (2019). Validation of the protein kinase PfCLK3 as a multistage cross-species malarial drug target. Science.

[136] Dorin-Semblat D., Demarta-Gatsi C., Hamelin R., Armand F., Carvalho T.G., Moniatte M., Doerig C. (2015). Malaria parasite infected erythrocytes secrete PfCK1, the *Plasmodium* homologue of the pleiotropic protein kinase casein kinase 1. PLoS ONE.

[137] Batty M.B., Schittenhelm R.B., Dorin-Semblat D., Doerig C., Garcia-Bustos J.F. (2020). Interaction of *Plasmodium falciparum* casein kinase 1 with components of host cell protein trafficking machinery. IUBMB Life.

[138] Reininger L., Wilkes J.M., Bourgade H., Miranda-Saavedra D., Doerig C. (2011). An essential Aurora-related kinase transiently associates with spindle pole bodies during *Plasmodium falciparum* erythrocytic schizogony. Mol. Microbiol..

[139] Carvalho T.G., Doerig C., Reininger L. (2013). Nima- and Aurora-related kinases of malaria parasites. Biochim. Biophys. Acta.

[140] Desoubzdanne D., Marcourt L., Raux R., Chevalley S., Dorin D., Doerig C., Valentin A., Ausseil F., Debitus C. (2008). Alisiaquinones and alisiaquinol, dual inhibitors of *Plasmodium falciparum* enzyme targets from a new caledonian deep water sponge. J. Nat. Prod..

[141] Laurent D., Jullian V., Parenty A., Knibiehler M., Dorin D., Schmitt S., Lozach O., Lebouvier N., Frostin M., Alby F. (2006). Antimalarial potential of xestoquinone, a protein kinase inhibitor isolated from a Vanuatu marine sponge *Xestospongia* sp.. Bioorg. Med. Chem..

[142] Lebouvier N., Jullian V., Desvignes I., Maurel S., Parenty A., Dorin-Semblat D., Doerig C., Sauvain M., Laurent D. (2009). Antiplasmodial activities of homogentisic acid derivative protein kinase inhibitors isolated from a Vanuatu marine sponge *Pseudoceratina* sp.. Mar. Drugs.

[143] Patel G., Roncal N.E., Lee P.J., Leed S.E., Erath J., Rodriguez A., Sciotti R.J., Pollastri M.P. (2014). Repurposing human Aurora kinase inhibitors as leads for anti-protozoan drug discovery. Medchemcomm.

[144] Brown J.R., Auger K.R. (2011). Phylogenomics of phosphoinositide lipid kinases: Perspectives on the evolution of second messenger signalling and drug discovery. BMC Evol. Biol..

[145] McNamara C.W., Lee M.C., Lim C.S., Lim S.H., Roland J., Simon O., Yeung B.K., Chatterjee A.K., McCormack S.L., Manary M.J. (2013). Targeting *Plasmodium* PI(4)K to eliminate malaria. Nature.

[146] Tawk L., Chicanne G., Dubremetz J.F., Richard V., Payrastre B., Vial H.J., Roy C., Wengelnik K. (2010). Phosphatidylinositol 3-phosphate, an essential lipid in *Plasmodium*, localizes to the food vacuole membrane and the apicoplast. Eukaryot Cell.

[147] Vaid A., Ranjan R., Smythe W.A., Hoppe H.C., Sharma P. (2010). PfPI3K, a phosphatidylinositol-3 kinase from *Plasmodium falciparum*, is exported to the host erythrocyte and is involved in hemoglobin trafficking. Blood.

[148] Mbengue A., Bhattacharjee S., Pandharkar T., Liu H., Estiu G., Stahelin R.V., Rizk S.S., Njimoh D.L., Ryan Y., Chotivanich K. (2015). A molecular mechanism of artemisinin resistance in *Plasmodium falciparum* malaria. Nature.

[149] Ibrahim M.A.A., Abdelrahman A.H.M., Hassan A.M.A. (2019). Identification of novel *Plasmodium falciparum* PI4KB inhibitors as potential anti-malarial drugs: Homology modeling, molecular docking and molecular dynamics simulations. Comput. Biol. Chem..

[150] Le Manach C., Gonzalez Cabrera D., Douelle F., Nchinda A.T., Younis Y., Taylor D., Wiesner L., White K.L., Ryan E., March C. (2014). Medicinal chemistry optimization of antiplasmodial imidazopyridazine hits from high throughput screening of a SoftFocus kinase library: Part 1. J. Med. Chem..

[151] Le Manach C., Paquet T., Gonzalez Cabrera D., Younis Y., Taylor D., Wiesner L., Lawrence N., Schwager S., Waterson D., Witty M.J. (2014). Medicinal chemistry optimization of antiplasmodial imidazopyridazine hits from high throughput screening of a softfocus kinase library: Part 2. J. Med. Chem..

[152] Zou B., Nagle A., Chatterjee A.K., Leong S.Y., Tan L.J., Sim W.L., Mishra P., Guntapalli P., Tully D.C., Lakshminarayana S.B. (2014). Lead optimization of imidazopyrazines: A new class of antimalarial with activity on *Plasmodium* liver stages. ACS Med. Chem. Lett..

[153] Gibhard L., Njoroge M., Paquet T., Brunschwig C., Taylor D., Lawrence N., Abay E., Wittlin S., Wiesner L., Street L.J. (2018). Investigating Sulfoxide-to-Sulfone Conversion as a Prodrug Strategy for a Phosphatidylinositol 4-Kinase Inhibitor in a Humanized Mouse Model of Malaria. Antimicrob. Agents Chemother..

[154] Le Manach C., Nchinda A.T., Paquet T., Gonzalez Cabrera D., Younis Y., Han Z., Bashyam S., Zabiulla M., Taylor D., Lawrence N. (2016). Identification of a Potential Antimalarial Drug Candidate from a Series of 2-Aminopyrazines by Optimization of Aqueous Solubility and Potency across the Parasite Life Cycle. J. Med. Chem..

[155] Younis Y., Douelle F., Feng T.S., Gonzalez Cabrera D., Le Manach C., Nchinda A.T., Duffy S., White K.L., Shackleford D.M., Morizzi J. (2012). 3,5-Diaryl-2-aminopyridines as a novel class of orally active antimalarials demonstrating single dose cure in mice and clinical candidate potential. J. Med. Chem..

[156] Younis Y., Douelle F., Gonzalez Cabrera D., Le Manach C., Nchinda A.T., Paquet T., Street L.J., White K.L., Zabiulla K.M., Joseph J.T. (2013). Structure-activity-relationship studies around the 2-amino group and pyridine core of antimalarial 3,5-diarylaminopyridines lead to a novel series of pyrazine analogues with oral in vivo activity. J. Med. Chem..

[157] Liang X., Jiang Z., Huang Z., Li F., Chen C., Hu C., Wang W., Hu Z., Liu Q., Wang B. (2020). Discovery of 6′-chloro-N-methyl-5′-(phenylsulfonamido)-[3,3′-bipyridine]-5-carboxamide (CHMFL-PI4K-127) as a novel *Plasmodium falciparum* PI(4)K inhibitor with potent antimalarial activity against both blood and liver stages of *Plasmodium*. Eur. J. Med. Chem..

[158] Paquet T., Le Manach C., Cabrera D.G., Younis Y., Henrich P.P., Abraham T.S., Lee M.C.S., Basak R., Ghidelli-Disse S., Lafuente-Monasterio M.J. (2017). Antimalarial efficacy of MMV390048, an inhibitor of *Plasmodium* phosphatidylinositol 4-kinase. Sci. Transl. Med..

[159] Sinxadi P., Donini C., Johnstone H., Langdon G., Wiesner L., Allen E., Duparc S., Chalon S., McCarthy J.S., Lorch U. (2020). Safety, tolerability, pharmacokinetics, and antimalarial activity of the novel *Plasmodium* phosphatidylinositol 4-kinase inhibitor MMV390048 in healthy volunteers. Antimicrob. Agents Chemother..

[160] Brunschwig C., Lawrence N., Taylor D., Abay E., Njoroge M., Basarab G.S., Le Manach C., Paquet T., Cabrera D.G., Nchinda A.T. (2018). UCT943, a next-generation *Plasmodium falciparum* PI4K inhibitor preclinical candidate for the treatment of malaria. Antimicrob. Agents Chemother..

[161] Chaudhary K.K., Gupta S.K., Mishra N. (2016). An In Silico Approach for Targeting *Plasmodium* Phosphatidylinositol 4-Kinase to Eradicate Malaria. Advanced Computing and Communication Technologies.

[162] Ren J.X., Gao N.N., Cao X.S., Hu Q.A., Xie Y. (2016). Homology modeling and virtual screening for inhibitors of lipid kinase PI(4)K from *Plasmodium*. Biomed. Pharm..

[163] Lucet I.S., Tobin A., Drewry D., Wilks A.F., Doerig C. (2012). *Plasmodium* kinases as targets for new-generation antimalarials. Future Med. Chem..

[164] Dorin D., Semblat J.P., Poullet P., Alano P., Goldring J.P., Whittle C., Patterson S., Chakrabarti D., Doerig C. (2005). PfPK7, an atypical MEK-related protein kinase, reflects the absence of classical three-component MAPK pathways in the human malaria parasite *Plasmodium falciparum*. Mol. Microbiol..

[165] Dorin-Semblat D., Sicard A., Doerig C., Ranford-Cartwright L., Doerig C. (2008). Disruption of the PfPK7 gene impairs schizogony and sporogony in the human malaria parasite *Plasmodium falciparum*. Eukaryot Cell.

[166] Zhang M., Wang C., Otto T.D., Oberstaller J., Liao X., Adapa S.R., Udenze K., Bronner I.F., Casandra D., Mayho M. (2018). Uncovering the essential genes of the human malaria parasite *Plasmodium falciparum* by saturation mutagenesis. Science.

[167] Philip N., Haystead T.A. (2007). Characterization of a UBC13 kinase in *Plasmodium falciparum*. Proc. Natl. Acad. Sci. USA.

[168] Schneider A.G., Mercereau-Puijalon O. (2005). A new Apicomplexa-specific protein kinase family: Multiple members in *Plasmodium falciparum*, all with an export signature. BMC Genom..

[169] Nunes M.C., Goldring J.P., Doerig C., Scherf A. (2007). A novel protein kinase family in *Plasmodium falciparum* is differentially transcribed and secreted to various cellular compartments of the host cell. Mol. Microbiol..

[170] Nunes M.C., Okada M., Scheidig-Benatar C., Cooke B.M., Scherf A. (2010). *Plasmodium falciparum* FIKK kinase members target distinct components of the erythrocyte membrane. PLoS ONE.

[171] Siddiqui G., Proellochs N.I., Cooke B.M. (2020). Identification of essential exported *Plasmodium falciparum* protein kinases in malaria-infected red blood cells. Br. J. Haematol..

[172] Kats L.M., Fernandez K.M., Glenister F.K., Herrmann S., Buckingham D.W., Siddiqui G., Sharma L., Bamert R., Lucet I., Guillotte M. (2014). An exported kinase (FIKK42) that mediates virulence-associated changes in *Plasmodium falciparum*-infected red blood cells. Int. J. Parasitol..

[173] Brandt G.S., Bailey S. (2013). Dematin, a human erythrocyte cytoskeletal protein, is a substrate for a recombinant FIKK kinase from *Plasmodium falciparum*. Mol. Biochem. Parasitol..

[174] Merckx A., Echalier A., Langford K., Sicard A., Langsley G., Joore J., Doerig C., Noble M., Endicott J. (2008). Structures of P. falciparum protein kinase 7 identify an activation motif and leads for inhibitor design. Structure.

[175] Bouloc N., Large J.M., Smiljanic E., Whalley D., Ansell K.H., Edlin C.D., Bryans J.S. (2008). Synthesis and in vitro evaluation of imidazopyridazines as novel inhibitors of the malarial kinase PfPK7. Bioorg. Med. Chem. Lett..

[176] Klein M., Diner P., Dorin-Semblat D., Doerig C., Grotli M. (2009). Synthesis of 3-(1,2,3-triazol-1-yl)- and 3-(1,2,3-triazol-4-yl)-substituted pyrazolo[3,4-d]pyrimidin-4-amines via click chemistry: Potential inhibitors of the *Plasmodium falciparum* PfPK7 protein kinase. Org. Biomol. Chem..

[177] Raphemot R., Eubanks A.L., Toro-Moreno M., Geiger R.A., Hughes P.F., Lu K.Y., Haystead T.A.J., Derbyshire E.R. (2019). *Plasmodium* PK9 Inhibitors Promote Growth of Liver-Stage Parasites. Cell Chem. Biol..

[178] Totzke J., Gurbani D., Raphemot R., Hughes P.F., Bodoor K., Carlson D.A., Loiselle D.R., Bera A.K., Eibschutz L.S., Perkins M.M. (2017). Takinib, a Selective TAK1 Inhibitor, Broadens the Therapeutic Efficacy of TNF-alpha Inhibition for Cancer and Autoimmune Disease. Cell Chem. Biol..

[179] Hodge C.D., Spyracopoulos L., Glover J.N.M. (2016). Ubc13: The Lys63 ubiquitin chain building machine. Oncotarget.

[180] Tewari R., Straschil U., Bateman A., Bohme U., Cherevach I., Gong P., Pain A., Billker O. (2010). The systematic functional analysis of *Plasmodium* protein kinases identifies essential regulators of mosquito transmission. Cell Host Microbe.

[181] Osman K.T., Lou H.J., Qiu W., Brand V., Edwards A.M., Turk B.E., Hui R. (2015). Biochemical characterization of FIKK8--A unique protein kinase from the malaria parasite *Plasmodium falciparum* and other apicomplexans. Mol. Biochem. Parasitol..

[182] Lin B.C., Harris D.R., Kirkman L.M.D., Perez A.M., Qian Y., Schermerhorn J.T., Hong M.Y., Winston D.S., Xu L., Brandt G.S. (2017). FIKK Kinase, a Ser/Thr Kinase Important to Malaria Parasites, Is Inhibited by Tyrosine Kinase Inhibitors. ACS Omega.

[183] Knight Z.A., Shokat K.M. (2005). Features of selective kinase inhibitors. Chem. Biol..

[184] Lin B.C., Harris D.R., Kirkman L.M.D., Perez A.M., Qian Y., Schermerhorn J.T., Hong M.Y., Winston D.S., Xu L., Lieber A.M. (2017). The anthraquinone emodin inhibits the non-exported FIKK kinase from *Plasmodium falciparum*. Bioorg. Chem..

[185] Yuan J., Johnson R.L., Huang R., Wichterman J., Jiang H., Hayton K., Fidock D.A., Wellems T.E., Inglese J., Austin C.P. (2009). Genetic mapping of targets mediating differential chemical phenotypes in *Plasmodium falciparum*. Nat. Chem. Biol..

